# Common dysregulated pathways in obese adipose tissue and atherosclerosis

**DOI:** 10.1186/s12933-016-0441-2

**Published:** 2016-08-26

**Authors:** V. Moreno-Viedma, M. Amor, A. Sarabi, M. Bilban, G. Staffler, M. Zeyda, T. M. Stulnig

**Affiliations:** 1 Christian Doppler Laboratory for Cardio-Metabolic Immunotherapy and Clinical Division of Endocrinology and Metabolism, Department of Medicine III, Medical University of Vienna, Waehringer Guertel 18-20, 1090 Vienna, Austria; 2 Department of Laboratory Medicine & Core Facility Genomics, Core Facilities, Medical University of Vienna, Vienna, Austria; 3 AFFiRiS AG, Vienna, Austria; 4 Department of Pediatrics and Adolescent Medicine, Clinical Division of Pediatric Pulmonology, Allergology and Endocrinology, Medical University of Vienna, Vienna, Austria

**Keywords:** Cardiovascular diseases, Diabetes mellitus, type 2, Insulin resistance, Atherosclerosis, Pathway analysis

## Abstract

**Background:**

The metabolic syndrome is becoming increasingly prevalent in the general population that is at simultaneous risk for both type 2 diabetes and cardiovascular disease. The critical pathogenic mechanisms underlying these diseases are obesity-driven insulin resistance and atherosclerosis, respectively. To obtain a better understanding of molecular mechanisms involved in pathogenesis of the metabolic syndrome as a basis for future treatment strategies, studies considering both inherent risks, namely metabolic and cardiovascular, are needed. Hence, the aim of this study was to identify pathways commonly dysregulated in obese adipose tissue and atherosclerotic plaques.

**Methods:**

We carried out a gene set enrichment analysis utilizing data from two microarray experiments with obese white adipose tissue and atherosclerotic aortae as well as respective controls using a combined insulin resistance-atherosclerosis mouse model.

**Results:**

We identified 22 dysregulated pathways common to both tissues with p values below 0.05, and selected inflammatory response and oxidative phosphorylation pathways from the Hallmark gene set to conduct a deeper evaluation at the single gene level. This analysis provided evidence of a vast overlap in gene expression alterations in obese adipose tissue and atherosclerosis with *Il7r*, *C3ar1*, *Tlr1*, *Rgs1* and *Semad4d* being the highest ranked genes for the inflammatory response pathway and *Maob*, *Bckdha*, *Aldh6a1*, *Echs1* and *Cox8a* for the oxidative phosphorylation pathway.

**Conclusions:**

In conclusion, this study provides extensive evidence for common pathogenic pathways underlying obesity-driven insulin resistance and atherogenesis which could provide a basis for the development of novel strategies to simultaneously prevent type 2 diabetes and cardiovascular disease in patients with metabolic syndrome.

**Electronic supplementary material:**

The online version of this article (doi:10.1186/s12933-016-0441-2) contains supplementary material, which is available to authorized users.

## Background

The metabolic syndrome is a worldwide public health challenge with a prevalence above 20 % within adults in Western societies [1]. This disorder is based on several factors including visceral obesity, hypertension, dyslipidemia and hyperglycemia conferring a fivefold increased risk for type 2 diabetes and twofold for cardiovascular disease compared to the non-affected population [2, 3]. A chronic low-grade inflammation in response to obesity originating from the white adipose tissue has been identified as the link between obesity, insulin resistance, type 2 diabetes and cardiovascular disease [4–7]. Due to the simultaneous occurrence of insulin resistance and atherosclerosis, a considerable number of pathogenic pathways might be shared in the development of both conditions. In the past decades traditional approaches have been confined to identify changes in the expression levels of individual genes between two different conditions, however the integration and comprehension of large amounts of data remained a challenge [8–10]. Recently several methods and bioinformatic tools have been developed to perform pathways analyses out of gene expression data enabling to manage, integrate and interpret them with a more holistic view and a biological meaning [11, 12]. Gene set enrichment analysis (GSEA) provides the possibility to compare data with different gene set databases of interest and reports group of genes associated with the same biological function or common pathways [12, 13]. Hence such analyses allow a more general picture on dysregulation compared to analyses focusing on individual genes.

Despite a number of investigations focusing on alterations leading to the development of either insulin resistance or atherosclerosis, there is no record in the literature systematically looking for dysregulated pathways common to insulin resistance and atherosclerosis in the same individual. Due to the concurring risk of type 2 diabetes and cardiovascular disease, the elucidation of dysregulated pathways in adipose tissue and atherosclerotic plaques should be based on an animal model that mirrors human disease by simultaneously developing adipose tissue inflammation/insulin resistance and atherosclerosis. Therefore, the aim of this study was the identification and analysis of common dysregulated pathways in obesity-induced adipose tissue inflammation and atherosclerotic plaque formation to elucidate interrelations in the concurrent development of type 2 diabetes and cardiovascular disease. Increasing our understanding on simultaneous dysregulation may indicate common molecular mechanisms that underlie type 2 diabetes and cardiovascular disease to facilitate novel preventive and therapeutic strategies in patients with metabolic syndrome. In this study we performed a pathway analysis using GSEA software with data from an own microarray experiment carried out with gonadal white adipose tissue (AT) and aortae (AO) samples from a combined insulin resistance/atherosclerosis mouse model established in our lab [14]. With this inbred mouse model, we identified common pathways in the onset of adipose tissue inflammation/insulin resistance and atherosclerosis taking advantage of the simultaneous development of both of them in individual mice while avoiding genetic variation. In conclusion, this study provides a highly valuable set of information which may be used by multiple researchers to generate hypotheses on the common development of insulin resistance and atherosclerosis.

## Methods

### Animals and diets

A combined insulin resistance/atherosclerosis mouse model established in our laboratory was used as described [14]. At 9 weeks of age, male LDL-receptor knockout mice (*Ldlr*^−*/*−^*)* were placed for 20 weeks either on normal chow (NC; V1126-000, Ssnif, Soest, Germany) or diabetogenic diet (DDC; D09071704, Research Diets Inc.) (Additional file 1: Table S1). Animals were sacrificed and AT and AO were collected and immediately snap frozen in liquid nitrogen. All mice were housed in a specific pathogen-free facility with a 12 h light/dark cycle. Mice had free access to food and water. The protocol fully complied with the guidelines on accommodation and care of animals formulated by the European Convention for the Protection of Vertebrate Animals Used for Experimental and Other Scientific Purposes and was approved by the local ethics committee for animal studies and the Austrian Federal Ministry for Science and Research.

### Atherosclerosis quantification

En-face staining was used to determine atherosclerotic plaque formation as described [15]. Briefly, after sacrificing the mice the thorax was opened and the aorta was removed and cleaned removing all fat and connective tissue. Subsequently, the aorta was excised 2 mm above aortic root and below iliac bifurcation, opened longitudinally, pinned to silicone plates with acupuncture needles (asia-med, Suhl, Germany) and fixed overnight in 4 % paraformaldehyde, 5 % sucrose, 20 μM EDTA (pH 7.4). Atherosclerotic plaques were stained with Sudan IV for 15 min and destained with 75 % ethanol. Pictures were taken with a Sony Z-1000 camera and atherosclerotic lesion area was assessed by a person blinded to the samples by using ImageJ software.

### Microarray analysis

The frozen tissue samples were homogenized in TRIzol^®^ reagent (Invitrogen/Life Technologies, Carlsbad, CA, USA) and processed based on manufacturer’s instructions for the RNA isolation. Total RNA (1 μg) was used for GeneChip analysis, for AT preparation six individual samples were used, whereas in the case of AO each of the three individual samples were pooled in three groups due to a limited quantity of material. Terminal labeled cDNA, hybridization to genome-wide Mouse Gene 2.0 ST Gene Chips and scanning of the arrays were carried out according to the manufacture’s indications (Affymetrix). Robust Multiarray Average (RMA) signal extraction, normalization and filtering were performed as described (http://www.bioconductor.org) [16, 17]. The data discussed in this publication have been deposited in NCBI’s Gene Expression Omnibus (TM. Stulnig et al. 2016) and are accessible through GEO Series accession number GSE76812 (http://www.ncbi.nlm.nih.gov/geo/query/acc.cgi?acc=GSE76812).

### Pathways analysis

For the GSEA analysis, output primary raw data from the AT or AO microarray experiments was set up as the expression data set in accordance with GSEA indications and uploaded to the software collectively with the phenotype labels, chip annotations and either Biocarta, KEGG, Reactome or Hallmark gene sets downloaded from the Molecular Signatures Database (MSigDB) from the GSEA website. Subsequently the program was run with 1000 permutations and gene set as a permutation type, obtaining all dysregulated pathways, their respective normalized enrichment score (NES) and the enrichment plot for each microarray experiment. Statistical significances were set at a nominal p < 0.05 and false discovery rate q < 25 %. According to the GSEA directions, the pathways upregulated by NC were taken as pathways downregulated by DDC.

Afterwards, the leading edge analysis function was executed to determine all those genes that significantly contribute to the dysregulation of the pathway of interest, also called leading genes. GSEA software was also used for perform the leading genes heat maps, where higher expression values are represented with red and lower expression values with dark blue. All Venn diagrams were made with the free access Venn Diagram Plotter from the Pacific Northwest National Laboratory.

### Statistical analyses

Data are given as mean ± SEM. Dietary treatment differences were estimated by unpaired two-tailed Student *t* test.

## Results

### Diabetogenic diet induces obesity and atherosclerosis in *Ldlr*^−*/*−^*mice*

**Fig. 1:**
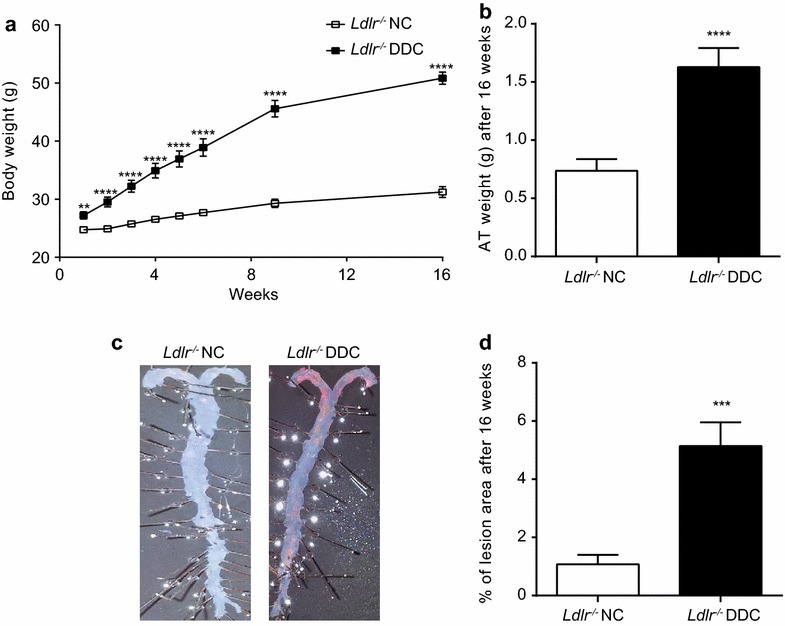
Diabetogenic diet induces obesity and atherosclerosis in Ldlr^−/−^ mice. Ldlr^−/−^ mice were placed on either diabetogenic diet (DDC) or normal chow (NC) for 16 weeks: **a** mean body weight during dietary treatment (n = 12); **b** mean gonadal white adipose tissue (AT) weight after 16 weeks on dietary treatment (n = 12); **c** representative images of en-face Sudan IV stained aortae after 16 weeks of indicated treatments; **d** atherosclerotic lesion quantification after 16 weeks of dietary treatment (n = 3)

Male *Ldlr*^−*/*−^ mice were fed DDC or NC following the combined cardiometabolic mouse model previously established and characterized in detail in our lab [14]. The key parameters of this model were reevaluated for the mice in this study. We observed a significantly higher body weight at all time points (Fig. 1a) as well an elevated final AT weight (Fig. 1 b) in *Ldlr*^−*/*−^ mice fed DDC for 16 weeks compared to those on NC animals. Atherosclerotic plaque formation as analyzed by en-face staining revealed markedly enhanced atherosclerotic lesions in *Ldlr*^−/−^ mice fed with DDC (Fig. 1c, d). Together these results point to the simultaneous development of considerable obesity and atherosclerosis in *Ldlr*^−/−^ mice fed with DDC used in this study, reflecting published findings in this mouse model.

### GSEA revealed common dysregulated pathways in AT and AO from obese mice

**Fig. 2:**
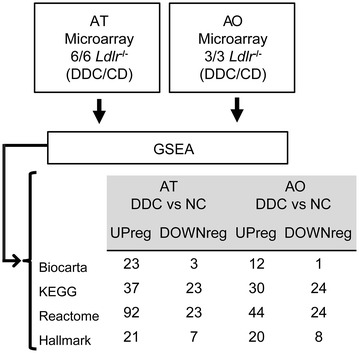
Study design and pathways GSEA results overview. The data from gonadal white adipose tissue (AT) and atherosclerotic aorta (AO) microarrays were analyzed by GSEA software with Biocarta, KEGG, Reactome or Hallmark as gene sets. Upregulated or downregulated pathways in AT or AO by the effect of the diabetogenic diet (DDC) are shown

**Fig. 3:**
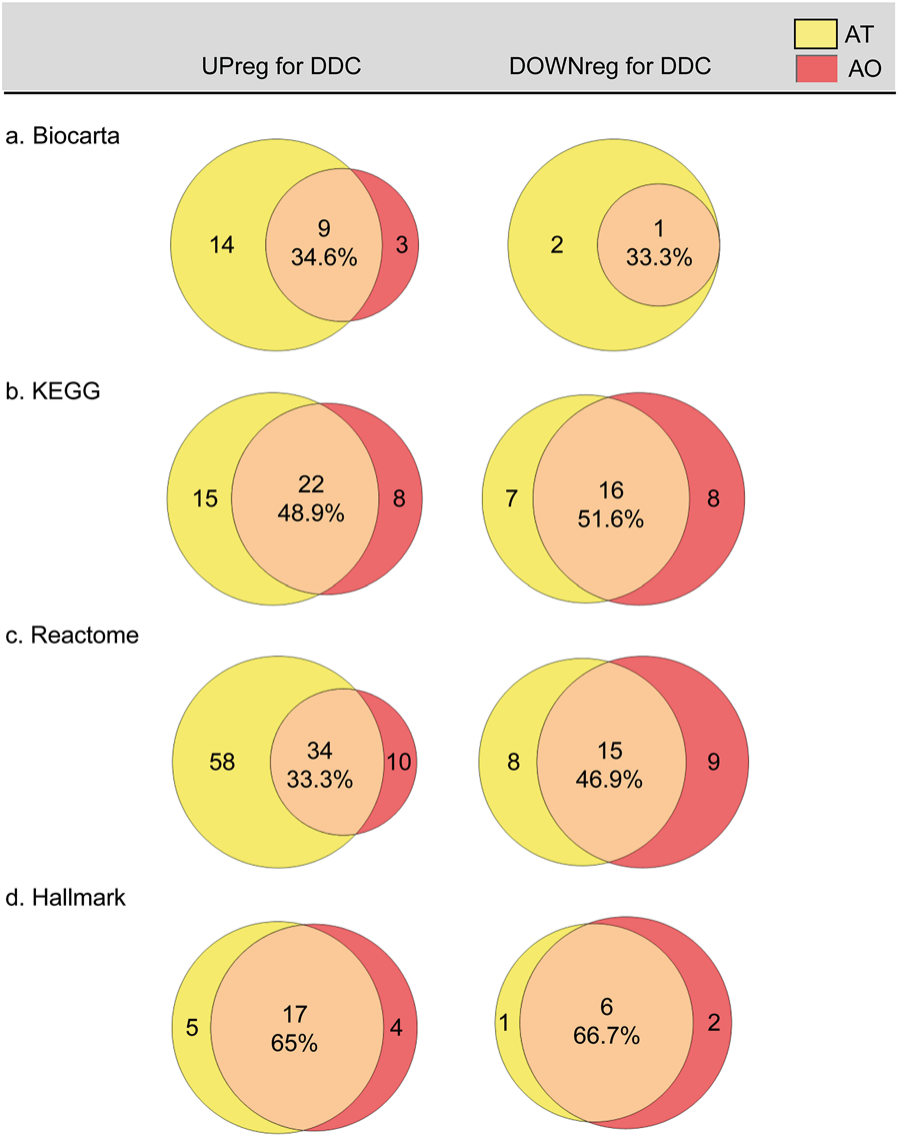
Venn diagrams plots for the dysregulated pathways according to GSEA. Upregulated or downregulated pathways for obese gonadal white adipose tissue (AT) and atherosclerotic aorta (AO) are shown in the Venn diagrams for each of the four analyses: **a** biocarta, **b** KEGG, **c** reactome, **d** Hallmark. Common pathways and percentage of overlap are included in the *central region* of each *diagram*

In this study, the output primary raw data from the AT or AO microarrays were uploaded into the GSEA with the aim to determine significantly dysregulated pathways in either tissue by the effect of the supplied diets (DDC or NC) and possible overlaps in terms of dysregulated pathways between both tissues. The analyses were compared with Biocarta, KEGG, Reactome or Hallmark as gene sets, obtaining upregulated pathways by DDC or NC for either AT or AO (Fig. 2). To identify common dysregulated pathways between AT and AO, we proceeded to match the upregulated or downregulated pathways for both tissues in each of the four analyses finding a considerable overlap in all the gene sets. The highest proportion of overlapping pathways was obtained for the Hallmark gene set (Fig. 3). Hence, the Hallmark gene set analysis was chosen for more comprehensive examinations, which allowed us to elucidate common pathways related to obesity induced AT inflammation and atherosclerosis.

Hallmark gene set involves many well characterized biological processes combining numerous pathways. The dysregulated pathways from the GSEA with the Hallmark gene set are shown in Table 1 and were ranked by AT microarray NES, the main statistic to evaluate gene set enrichment. Several well-known as well as novel pathways potentially involved in type 2 diabetes and atherosclerosis were dysregulated in AT and AO from obese and atherosclerotic animals, respectively, compared with healthy counterparts. Upregulation of inflammatory genes with positive NES occurred in both AT and AO in obesity and atherosclerosis. With respect to a pathway downregulated in obese adipose tissue (negative NES), we selected the oxidative phosphorylation pathway due to experimental and clinical evidence linking mitochondrial alterations to type 2 diabetes and atherosclerosis.

**Table 1:** Dysregulated pathways from obese adipose tissue and atherosclerotic aortae after Hallmark-GSEA analysis

DP^a^		NES^d^		NOM p-Val^e^		FDR^f^		IRP^g^	MRP^h^	Name
AT^b^	AO^c^	AT	AO	AT	AO	AT	AO			
*x*	*x*	*2.43*	*1.35*	*<0.001*	*0.047*	*0.000*	*0.098*			*E2F targets*
*x*	*x*	*2.39*	*1.74*	*<0.001*	*<0.001*	*0.000*	*0.004*			*G2* *M checkpoint*
*x*	*x*	*2.15*	*2.27*	*<0.001*	*<0.001*	*0.000*	*0.000*	*x*		*Allograft rejection*
*x*	*x*	*2.12*	*2.33*	*<0.001*	*<0.001*	*0.000*	*0.000*	*x*		*Inflammatory response*
*x*	*x*	*2.08*	*1.99*	*<0.001*	*<0.001*	*0.000*	*0.001*	*x*		*IL6 JAK STAT3 signaling*
*x*	*x*	*1.99*	*1.90*	*<0.001*	*<0.001*	*0.000*	*0.001*			*Epithelial mesenchymal transition*
*x*	*x*	*1.97*	*1.97*	*<0.001*	*<0.001*	*0.000*		*x*		*TNFA signaling via NFKB*
*x*	*x*	*1.88*	*1.75*	*<0.001*	*<0.001*	*0.000*				*Mitotic spindle*
*x*	*x*	*1.86*	*2.10*	*<0.001*	*<0.001*	*0.000*	*0.000*			*Complement*
*x*	*x*	*1.86*	*2.10*	*<0.001*	*<0.001*	*0.000*		*x*		*Kras signaling up*
*x*	*x*	*1.81*	*1.41*	*0.002*	*0.045*	*0.001*				*Protein secretion*
*x*	*x*	*1.72*	*1.89*	*<0.001*	*<0.001*	*0.004*	*0.001*			*Apoptosis*
x	x	*1.63*	*–1.34*	*0.001*	*0.026*	*0.008*	*0.091*		x	*MTORC1 signaling*
*x*	*x*	*1.60*	*2.35*	*0.001*	*<0.001*	*0.011*	*0.000*	*x*		*Interferon gamma response*
*x*	*x*	*1.58*	*1.61*	*0.021*	*0.021*	*0.013*	*0.011*		*x*	*Angiogenesis*
*x*	*x*	*1.50*	*1.48*	*0.006*	*0.012*	*0.027*	*0.035*			*Coagulation*
x		1.48		0.012		0.030				Unfolded protein response
x		1.43		0.015		0.047				MYC targets V1
x		1.41		0.024		0.051			x	Androgen response
x		1.36		0.027		0.073		x		IL2 STAT5 signaling
*x*	*x*	*1.34*	*1.68*	*0.024*	*<0.001*	*0.084*				*P53 pathway*
*x*	*x*	*−1.45*	*−1.72*	*0.016*	*<0.001*	*0.036*	*0.004*		*x*	*Peroxisome*
x		*−1.56*		*<0.001*		*0.000*				*Myogenesis*
*x*	*x*	*−1.65*	*−2.14*	*<0.001*	*<0.001*	*0.000*	*0.000*		*x*	*Bile acid metabolism*
*x*	*x*	*−1.86*	*–1.46*	*<0.001*	*0.003*	*0.000*	*0.038*			*Xenobiotic metabolism*
*x*	*x*	*–2.17*	*–2.40*	*<0.001*	*<0.001*	*0.000*	*0.000*		*x*	*Fatty acid metabolism*
*x*	*x*	*–2.44*	*−2.15*	*<0.001*	*<0.001*	*0.000*	*0.000*		*x*	*Oxidative phosphorylation*
*x*	*x*	*−3.06*	*−2.38*	*<0.001*	*<0.001*	*0.000*	*0.000*		*x*	*Adipogenesis*
	x		2.08		<0.001		0.000	x		Interferon alpha response
	x		1.72		<0.001		0.005			Heme metabolism
	x		1.63		0.001		0.010			Apical junction
	x		1.50		0.007		0.031		x	Hypoxia
	x		–1.77	<0.001			0.002			Spermatogenesis

Commonly dysregulated pathways in AT and AO are shown in italics

Dysregulated pathways are sorted by descending NES in AT

^a^Dysregulated pathways

^b^Obese white adipose tissue

^c^Atherosclerosic aortae

^d^Normalized enrichment score

^e^Nominal p value

^f^False discovery rate

^g^Inflammation related pathways

^h^Metabolism related pathways

### Single gene analysis of inflammatory response and oxidative phosphorylation pathways

**Fig. 4:**
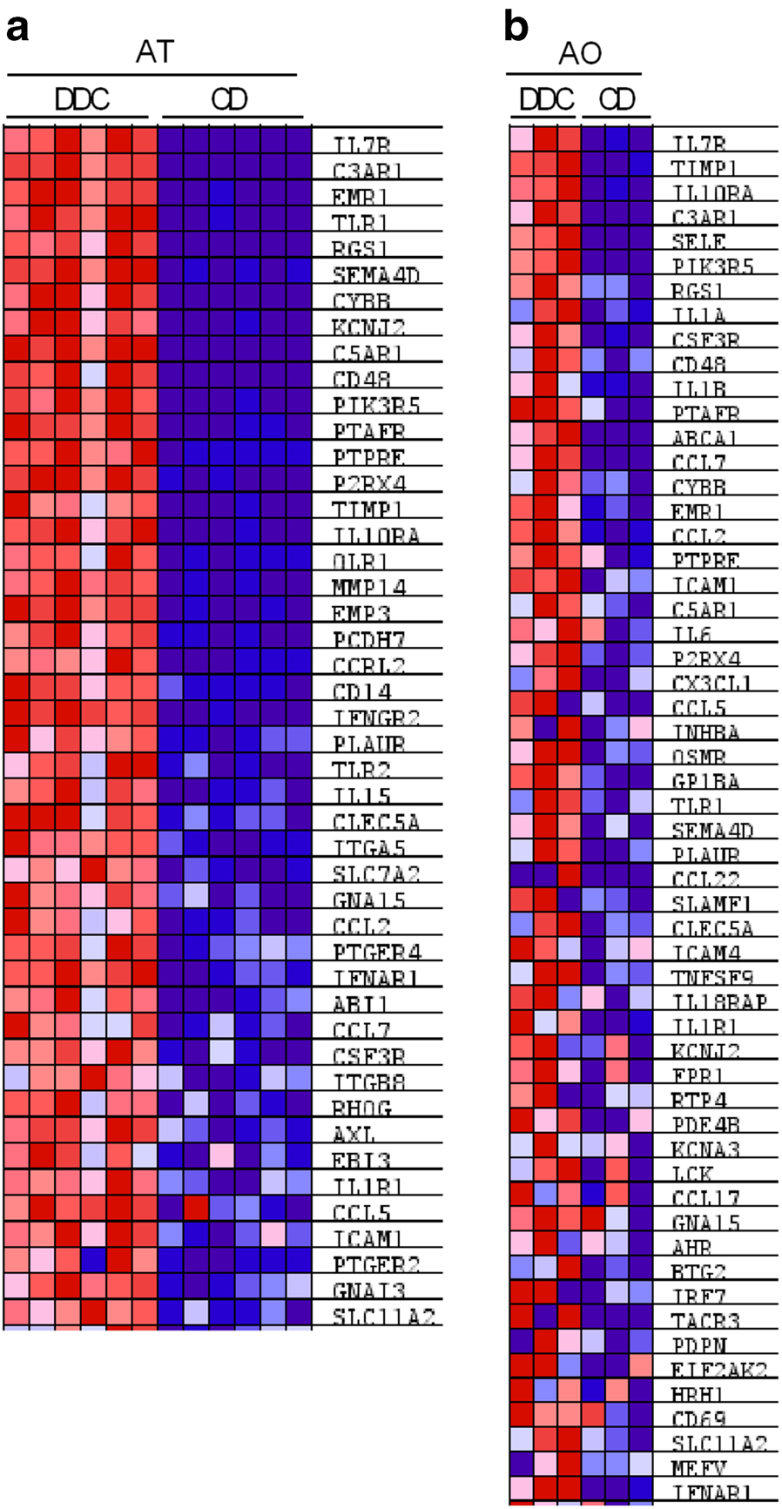
Leading genes heat map expression for the inflammatory response pathway in AT and AO. Leading genes of the inflammatory response pathway in: **a** gonadal white adipose tissue (AT) and **b** aorta (AO) are represented in the heatmaps. Upregulated genes are represented in* red* and downregulated genes are represented in *blue*

**Fig. 5:**
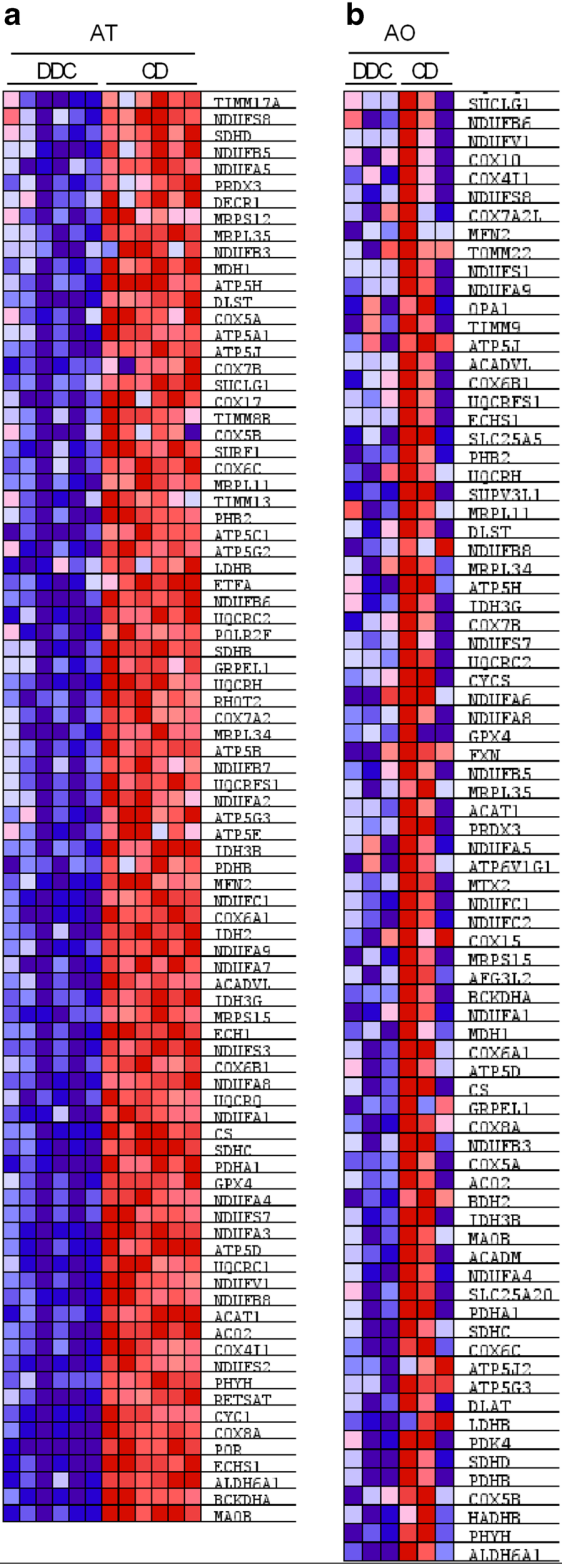
Leading genes heat map expression for the oxidative phosphorylation pathway in AT and AO. Leading genes of the oxidative phosphorylation pathway in: **a** gonadal white adipose tissue (AT) and **b** aorta (AO) are represented in the heatmaps. Upregulated genes are represented in red and downregulated genes are represented in *blue*

To investigate changes at the single gene level involved in the inflammatory response and oxidative phosphorylation pathways, we first proceeded with a leading edge analysis, which allowed us to identify those genes that are significantly affecting the dysregulation of each pathway, called leading genes. The leading genes are represented in the heatmaps (Figs. 4, 5) and in the pathway enrichment plots (Additional files 2, 3). The enrichment score that gives an idea about the overall regulation of the pathway is represented by the score at the peak of the enrichment plot. We carried out a leading edge analysis for the inflammatory response pathway in obese adipose tissue (Fig. 4a) and in atherosclerotic aortae (Fig. 4b) as well as for the oxidative phosphorylation pathways also in obese adipose tissue (Fig. 5a) and in atherosclerotic aortae (Fig. 5b). For the obese adipose tissue, a definite leading genes regulation profile was observed in all the samples in both analyzed pathways, denoted by the clear color patterns in the heatmaps (Figs. 4a, 5a). In contrast to the AT results, the leading gene regulation profile either in the inflammatory response or in the oxidative phosphorylation pathways was not quite homogenous in all the AO samples (Figs. 4b, 5b). Nevertheless, the oxidative phosphorylation pathway was clearly downregulated in obese/atherosclerotic animals as shown in Table 1, heatmaps and enrichment plots.

**Fig. 6:**
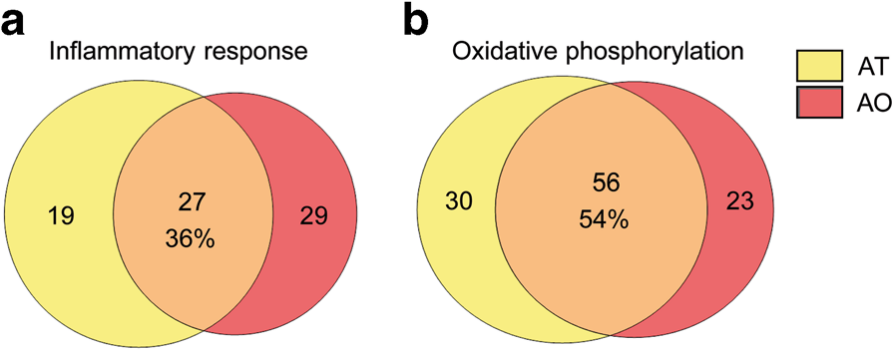
Venn diagrams plot of leading genes involved in the enrichment score of inflammatory response and phosphorylation pathways. Percentage of the overlap between the leading genes from the inflammatory response (**a**) and oxidative phosphorylation (**b**) pathways in obese white adipose tissue (AT) and in atherosclerotic aortae (AO)

We next tested for a possible overlap between the leading genes of each pathway in AT and AO. The percentage of matched leading genes from AT and AO in the inflammatory response pathway was 36 % (Fig. 6a), while it was even 54 % in oxidative phosphorylation (Fig. 6b). Additionally, the lists of common leading genes between both tissues for the inflammatory response pathway (Table 2) or oxidative phosphorylation pathway (Table 3) were obtained together with the corresponding rank metric score per gene which represents their importance in the dysregulation of the pathway with the uppermost listed genes being the most influential in the pathway in AT (Upregulated, Table 2; Downregulated, Table 3). For the inflammatory response pathway *Il7r*, *C3ar1*, *Tlr1*, *Rgs1* and *Semad4d* were the highest ranked genes. Among the most highly ranked in the oxidative phosphorylation pathway were *Maob*, *Bckdha*, *Aldh6a1*, *Echs1* and *Cox8a*. Non-overlapping genes regulated solely in AT or AO for inflammatory response and oxidative phosphorylation pathways are listed in the Additional file 1: Tables S2, S3, respectively. In addition, the common leading genes of the other pathways dysregulated in both analyzed tissues that have not been further analyzed in this study are presented in the Additional file 1: Table S4.

**Table 2:** Common genes from obese adipose tissue and atherosclerotic aortae involved in the inflammatory response pathway

Rank metric score		Gene symbol	Gene name
AT^a^	AO^b^		
4.554	2.000	*Il7r*	*Interleukin 7 receptor*
3.713	2.000	*C3ar1*	*Complement component 3a receptor 1*
3.652	0.928	*Emr1*	*Adhesion G protein*-*coupled receptor E1*
3.489	0.579	*Tlr1*	*Toll*-*like receptor 1*
3.298	1.000	*Rgs1*	*Regulator of G*-*protein signalling 1*
3.285	0.576	*Semad4d*	*Semaphorin 4D*
3.205	0.940	*Cybb*	*Cytochrome b*-*245 heavy chain*
3.175	0.445	*Kcnj2*	*Inward rectifier potassium channel 2*
3.111	0.779	*C5ar1*	*C5a anaphylatoxin chemotactic receptor 1*
3.037	1.000	*Cd48*	*CD48* *antigen*
2.961	1.000	*Pik3r5*	*Phosphoinositide*-*3*-*kinase, regulatory subunit 5, p101*
2.626	0.992	*Ptafr*	*Platelet*-*activating factor receptor*
2.422	0.894	*Ptpre*	*Protein tyrosine phosphatase, receptor type, E*
2.364	0.716	*P2rx4*	*Purinergic receptor P2X, ligand*-*gated ion channel, 4*
2.346	2.000	*Timp1*	*Tissue inhibitor of metalloproteinase 1*
2.197	2.000	*Il10ra*	*Interleukin 10 receptor, alpha*
1.692	0.519	*Plaur*	*Plasminogen activator, urokinase receptor*
1.594	0.496	*Clec5a*	*C*-*type lectin domain family 5, member a*
1.535	0.361	*Gna15*	*Guanine nucleotide binding protein, alpha 15*
1.452	0.922	*Ccl2*	*Chemokine (C*–*C motif) ligand 2*
1.303	0.273	*Ifnar1*	*Interferon (alpha and beta) receptor 1*
1.129	0.957	*Ccl7*	*Chemokine (C*–*C motif) ligand 7*
1.090	1.000	*Csf3r*	*Colony stimulating factor 3 receptor (granulocyte)*
0.921	0.460	*Il1r1*	*Interleukin 1 receptor, type I*
0.905	0.697	*Ccl5*	*Chemokine (C*–*C motif) ligand 5*
0.882	0.782	*Icam1*	*Intercellular adhesion molecule 1 (CD54), human rhinovirus receptor*
0.806	0.277	*Slc11a2*	*Solute carrier family 11, member 2*

Dysregulated genes are sorted by descending rank metric score in AT

^a^Obese gonadal white adipose tissue

^b^Atherosclerotic aortae

**Table 3:** Common genes from obese adipose tissue and atherosclerotic aortae involved in the oxidative phosphorylation pathway

Rank metric score		Gene symbol	Gene name
AT^a^	AO^b^		
−2.2950	−0.4060	*Maob*	*Monoamine oxidase B*
−2.0570	−0.3260	*Bckdha*	*Branched chain keto acid dehydrogenase E1, alpha polypeptide*
−2.0250	−0.8910	*Aldh6a1*	*Aldehyde dehydrogenase 6 family, member A1*
−2.0020	−0.2060	*Echs1*	*Enoyl CoA hydratase, short chain, 1, mitochondrial*
−1.7080	−0.3710	*Cox8a*	*Cytochrome c oxidase subunit 8a*
−1.4980	−0.7180	*Phyh*	*Phytanoyl*–*CoA hydroxylase*
−1.4240	−0.1680	*Cox4i1*	*Cytochrome c oxidase subunit IV isoform 1*
−1.3880	−0.3800	*Aco2*	*Aconitase 2, mitochondrial*
−1.3400	−0.2640	*Acat1*	*Acetyl*-*Coenzyme A acetyltransferase 1*
−1.3350	−0.2220	*Ndufb8*	*NADH dehydrogenase (ubiquinone) 1 beta subcomplex, 8*
−1.3200	−0.1620	*Ndufv1*	*NADH dehydrogenase (ubiquinone) flavoprotein 1*
−1.3060	−0.3470	*Atp5d*	*ATP synthase, H*+ *transporting, mitochondrial F1 complex, delta subunit*
−1.2750	−0.2330	*Ndufs7*	*NADH dehydrogenase (ubiquinone) Fe*–*S protein 7*
−1.2670	−0.4190	*Ndufa4*	*NADH dehydrogenase (ubiquinone) 1 alpha subcomplex, 4*
−1.2370	−0.2430	*Gpx4*	*Glutathione peroxidase 4*
−1.2260	−0.4390	*Pdha1*	*Pyruvate dehydrogenase E1 alpha 1*
−1.2060	−0.4440	*Sdhc*	*Succinate dehydrogenase complex, subunit C, integral membrane protein*
−1.2030	−0.3650	*Cs*	*Citrate synthase*
−1.1660	−0.3270	*Ndufa1*	*NADH dehydrogenase (ubiquinone) 1 alpha subcomplex, 1*
−1.1510	−0.2410	*Ndufa8*	*NADH dehydrogenase (ubiquinone) 1 alpha subcomplex, 8*
−1.1450	−0.1990	*Cox6b1*	*Cytochrome c oxidase subunit Vib polypeptide 1*
−1.1290	−0.3140	*Mrps15*	*Mitochondrial ribosomal protein S15*
−1.1150	−0.2280	*Idh3* *g*	*Isocitrate dehydrogenase 3 (NAD*+*) gamma*
−1.0870	−0.1990	*Acadvl*	*Acyl*-*Coenzyme A dehydrogenase, very long chain*
−1.0820	−0.1850	*Ndufa9*	*NADH dehydrogenase (ubiquinone) 1 alpha subcomplex, 9*
−1.0620	−0.3370	*Cox6a1*	*Cytochrome c oxidase subunit VIa polypeptide 1*
−1.0570	−0.2870	*Ndufc1*	*NADH dehydrogenase (ubiquinone) 1, subcomplex unknown, 1*
−1.0470	−0.1760	*Mfn2*	*Mitofusin 2*
−1.0230	−0.5940	*Pdhb*	*Pyruvate dehydrogenase (lipoamide) beta*
−1.0140	−0.3930	*Idh3b*	*Isocitrate dehydrogenase 3 (NAD*+*) beta*
−0.9750	−0.5050	*Atp5g3*	*ATP synthase, H* + *transporting, mitochondrial F0 complex, subunit C3 (subunit 9)*
−0.9440	−0.1990	*Uqcrfs1*	*Ubiquinol*-*cytochrome c reductase, Rieske iron*-*sulfur polypeptide 1*
−0.9060	−0.2240	*Mrpl34*	*Mitochondrial ribosomal protein L34*
−0.8870	−0.2150	*Uqcrh*	*Ubiquinol*-*cytochrome c reductase hinge protein*
−0.8840	−0.3650	*Grpel1*	*GrpE*−*like 1, mitochondrial*
−0.8440	−0.2340	*Uqcrc2*	*Ubiquinol*-*cytochrome c reductase core protein 2*
−0.8300	−0.1600	*Ndufb6*	*NADH dehydrogenase (ubiquinone) 1 beta subcomplex, 6*
−0.8010	−0.5270	*Ldhb*	*Lactate dehydrogenase B*
−0.7460	−0.2100	*Phb2*	*Prohibitin 2*
−0.7320	−0.2170	*Mrpl11*	*Mitochondrial ribosomal protein L11*
−0.7270	−0.4530	*Cox6c*	*Cytochrome c oxidase subunit VIc*
−0.7160	−0.6190	*Cox5b*	*Cytochrome c oxidase subunit Vb*
−0.7090	−0.1590	*Suclg1*	*Succinate*-*CoA ligase, GDP*-*forming, alpha subunit*
−0.7080	−0.2300	*Cox7b*	*Cytochrome c oxidase subunit VIIb*
−0.7030	−0.1930	*Atp5j*	*ATP synthase, H*+ *transporting, mitochondrial F0 complex, subunit F*
−0.6970	−0.3770	Cox5a	Cytochrome c oxidase subunit Va
−0.6920	−0.2190	*Dlst*	*Dihydrolipoamide S*-*succinyltransferase*
−0.6890	−0.2280	*Atp5* *h*	*ATP synthase, H*+ *transporting, mitochondrial F0 complex, subunit D*
−0.6880	−0.3350	*Mdh1*	*Malate dehydrogenase 1, NAD (soluble)*
−0.6860	−0.3760	*Ndufb3*	*NADH dehydrogenase (ubiquinone) 1 beta subcomplex, 3*
−0.6670	−0.2560	*Mrpl35*	*Mitochondrial ribosomal protein L35*
−0.6340	−0.2650	*Prdx3*	*Peroxiredoxin 3*
−0.6270	−0.2690	*Ndufa5*	*NADH dehydrogenase (ubiquinone) 1 alpha subcomplex, 5*
−0.6050	−0.2480	*Ndufb5*	*NADH dehydrogenase (ubiquinone) 1 beta subcomplex, 5*
−0.5830	−0.5650	*Sdhd*	*Succinate dehydrogenase complex, subunit D, integral membrane protein*
−0.5760	−0.1720	*Ndufs8*	*NADH dehydrogenase (ubiquinone) Fe*–*S protein 8*

Dysregulated genes are sorted by descending rank metric score in AT

^a^Obese gonadal white adipose tissue

^b^Atherosclerotic aortae

## Discussion

Type 2 diabetes and cardiovascular disease are common risks inherent with presence of metabolic syndrome and are triggered by insulin resistance and atherosclerosis, respectively. Several immunological and metabolic changes in serum levels from factors such as TNF-α [18], IL-6 [19], plasminogen activator inhibitor (PAI-1) [20], C-reactive protein (CRP) and fibrinogen [21], have been reported to be altered during insulin resistance and adipose tissue inflammation, both consequences of obesity, as well as atherosclerotic plaque formation during dyslipidemias [22, 23]. Even though diverse studies have been carried out to determine common variations at gene or protein levels between different metabolic tissues during obesity, the common pathways involved in the simultaneous development of adipose tissue inflammation and atherosclerosis remained elusive. Identification of common dysregulated pathways, however, could be a key in the development of both conditions and provide a chance for novel strategies to simultaneously prevent type 2 diabetes and cardiovascular disease in subjects at risk, i.e. those with metabolic syndrome [24, 25]. Pathway-based analysis is a powerful tool that allows to detect changes at a higher biological level than individual genes or molecules, complementing single gene level approaches with biological interactions, and thus contributing to a better understanding of the complexity of diseases [13]. Taking this together with the high reliability of the microarray technology nowadays [26–28], the pathways analyses based on microarray data can be considered as remarkably robust studies at the meta level. There are several publications showing single pathways to be involved in type 2 diabetes [29, 30] or atherosclerosis [31–33], such as focal adhesion [34], angiotensin II-NFκB [35], electron carrier activity, PPAR signaling and protein secretion [36]. However, in this study, we combined adipose tissue inflammation and atherosclerosis, in order to identify common dysregulated pathways in a systematical and unbiased manner, merging own and published data and applying different bioinformatic approaches.

Initially, we took advantage of a recently established combined insulin resistance/atherosclerosis inbred mouse model and analyzed microarray raw data from AT and AO with the GSEA software. Using four different gene sets, Biocarta, KEGG, Reactome and Hallmark (Fig. 2) we obtained considerably overlaps of dysregulated pathways in each of the four analyses. We choose Hallmark results to conduct a deeper look. However, to consider data from Biocarta, KEGG and Reactome analyses remains an interesting aim for further studies (Fig. 3).

Interestingly, the Hallmark analysis results included novel as well as already described common dysregulated pathways related with insulin resistance and atherosclerosis such as inflammatory response, IL6 JAK STAT3 signaling, TNFA signaling via NFKB, interferon gamma response, fatty acid metabolism, oxidative phosphorylation and adipogenesis (Table 1). This list of pathways could be of a great tool for researchers who are interested in the common mechanisms behind adipose tissue inflammation and atherosclerosis, since common genes enclosed in the pathways could be new targets to investigate mechanism as well as possible future treatments.

After evaluation of the common pathways altered in both metabolic processes, we selected the inflammatory and oxidative phosphorylation pathways as representatives of up- and down-regulated pathways, respectively, due to the importance of the involved genes in the pathogenesis of type 2 diabetes and atherosclerosis [37–41]. We could show a clear expression pattern for all genes that contribute to the dysregulation of the pathway in each tissue (Figs. 4, 5). The upregulation of the inflammatory response pathway in inflamed adipose tissue as well as in atherosclerotic aorta in the animal model used in this study complements the recent findings showing after a long term high fat diet (16 weeks) that the homeostasis of the immune system is altered from a physiological immune response to a pathological state [42]. Moreover, the down-regulation of the oxidative phosphorylation pathway in AT supports the observations indicating decreased expression of the genes implicated in the mitochondria electron chain in visceral adipose tissue in type 2 diabetes [39]. However, our study shows the down-regulation of the oxidative phosphorylation pathway also in the atherosclerotic aorta. Additionally we also identified a considerable number of common genes between both analyzed tissues that significantly contribute to the dysregulation of the inflammatory response or the oxidative phosphorylation pathways and could be potentially involved in the pathogenesis of both type 2 diabetes and cardiovascular disease (Fig. 6; Tables 2, 3).

A large amount of studies have been performed to identify potential treatment strategies for multifactorial diseases which are caused by a collective dysregulation of many genes. Recently, identification of dysregulated pathways was proposed as possible biomarkers for such disorders [43, 44]. This study conferred a strong evidence of similarities on the pathogenesis between insulin resistance and atherosclerosis, which could support diagnostic processes and drug design in a more effective manner facilitating a more personalized medicine in patients with metabolic syndrome.

## Conclusion

In conclusion, we describe analogies at the pathway as well as individual gene level between obese adipose tissue and atherosclerotic aortae potentially to be considered as a basis to achieve novel therapeutic approaches for the simultaneous prevention of type 2 diabetes and atherosclerotic cardiovascular disease.

## Additional files

10.1186/s12933-016-0441-2 Additional tables.
10.1186/s12933-016-0441-2 Enrichment plots for inflammatory response pathway in AT and AO. The enrichment plots for inflammatory response pathway in: (a) obese white adipose tissue (AT) and (b) atherosclerotic aorta (AO) are shown, representing at the top the enrichment score and the leading edge subset, at the middle the genes that appear in the rank list and in the bottom the ranking metric that measures the correlation between the gene expression and the phenotype.
10.1186/s12933-016-0441-2 Enrichment plots for oxidative phosphorylation pathway in AT and AO. The enrichment plots for oxidative phosphorylation pathway in: (a) obese gonadal white adipose tissue (AT) and (b) atherosclerotic aorta (AO) are shown, representing at the top the enrichment score and the leading edge subset, at the middle the genes that appear in the rank list and in the bottom the ranking metric that measures the correlation between the gene expression and the phenotype.

## Abbreviations

AO: aortae
AT: gonadal white adipose tissue
DDC: diabetogenic diet
FDR: false discovery rate
GSEA: gene set enrichment analysis
IRP: inflammation related pathways
*Ldlr*^−*/*−^: lDL-receptor knockout mice
MRP: metabolism related pathways
NC: normal chow diet
NES: normalized enrichment score
