# Supplementary material for: Common dysregulated pathways in obese adipose tissue and atherosclerosis
Source: Cardiovasc Diabetol. 2016 Aug 26;15(1):120. doi: 10.1186/s12933-016-0441-2 (PMC5000404; doi:10.1186/s12933-016-0441-2)
Supplement: Supplementary file 1 — 10.1186/s12933-016-0441-2 Additional tables. [file 12933_2016_441_MOESM1_ESM.doc]

**Additional File 1. Table S1. Macronutrient content of the normal and diabetogenic rodent chows used in the study.**

|  | **Normal chow (NC)** | | **Diabetogenic chow (DDC)** | |
| --- | --- | --- | --- | --- |
|  | **Content (%)** | **Energy (%)** | **Content (%)** | **Energy (%)** |
| **Proteins** | 22.0 | 26.0 | 20.5 | 15.0 |
| **Carbohydrates** | 52.7 | 61.0 | 38.1 | 28.0 |
| **Fat** | 4.8 | 13.0 | 35.8 | 58.0 |
| **Cholesterol** | - | - | 0.15 | - |

**Additionl File 1. Table S2. Non-common genes from obese AT and atherosclerotic AO** involved in the inflammatory response pathway.

| **Tissue** | | **Rank metric score** | | **Gene symbol** | **Gene name** |
| --- | --- | --- | --- | --- | --- |
| **AT**^A^ | **AO**^B^ | **AT**^A^ | **AO**^B^ |
| x |  | 2.193 | - | *Olr1* | *Oxidised low density lipoprotein (lectin-like) receptor 1* |
| x |  | 2.164 | - | *Mmp14* | *Matrix metallopeptidase 14 (membrane-inserted)* |
| x |  | 2.161 | - | *Emp3* | *Epithelial membrane protein 3* |
| x |  | 2.023 | - | *Pcdh7* | *Protocadherin 7* |
| x |  | 1.931 | - | *Ccrl2* | *Chemokine (C-C motif) receptor-like 2* |
| x |  | 1.910 | - | *Cd14* | *CD14 antigen* |
| x |  | 1.724 | - | *Ifngr2* | *Interferon gamma receptor 2* |
| x |  | 1.626 | - | *Tlr2* | *Toll-like receptor 2* |
| x |  | 1.613 | - | *Il15* | *Interleukin 15* |
| x |  | 1.594 | - | *Itga5* | *Integrin alpha 5 (fibronectin receptor alpha)* |
| x |  | 1.585 | - | *Slc7a2* | *Solute carrier family 7 (cationic amino acid transporter, y+ system), member 2* |
| x |  | 1.345 | - | *Ptger4* | *Prostaglandin E receptor 4 (subtype EP4)* |
| x |  | 1.294 | - | *Abi1* | *Abl-interactor 1* |
| x |  | 1.059 | - | *Itgb8* | *Integrin, beta 8* |
| x |  | 1.048 | - | *Rhog* | *Ras homolog gene family, member G* |
| x |  | 1.002 | - | *Axl* | *AXL receptor tyrosine kinase* |
| x |  | 0.976 | - | *Ebi3* | *Epstein-Barr virus induced gene 3* |
| x |  | 0.866 | - | *Ptger2* | *Prostaglandin E receptor 2 (subtype EP2)* |
| x |  | 0.811 | - | *Gnai3* | *Guanine nucleotide binding protein (G protein), alpha inhibiting 3* |
|  | x | - | 2.000 | *Sele* | *Selectin, endothelial cell* |
|  | x | - | 1.000 | *Il1a* | *Interleukin 1, alpha* |
|  | x | - | 1.000 | *Il1b* | *Interleukin 1, beta* |
|  | x | - | 0.989 | *Abca1* | *ATP-binding cassette, sub-family A (ABC1), member 1* |
|  | x | - | 0.754 | *Il6* | *Interleukin 6* |
|  | x | - | 0.705 | *Cx3cl1* | *Chemokine (C-X3-C motif) ligand 1* |
|  | x | - | 0.628 | *Inhba* | *Inhibin beta A* |
|  | x | - | 0.613 | *Osmr* | *Oncostatin M receptor* |
|  | x | - | 0.587 | *Gp1ba* | *Glycoprotein Ib, alpha polypeptide* |
|  | x | - | 0.517 | *Ccl22* | *Chemokine (C-C motif) ligand 22* |
|  | x | - | 0.505 | *Slamf1* | *Signaling lymphocytic activation molecule family member 1* |
|  | x | - | 0.484 | *Icam4* | *Intercellular adhesion molecule 4 (Landsteiner-Wiener blood group)* |
|  | x | - | 0.467 | *Tnfsf9* | *Tumor necrosis factor (ligand) superfamily, member 9* |
|  | x | - | 0.465 | *Il18rap* | *Interleukin 18 receptor accessory protein* |
|  | x | - | 0.398 | *Fpr1* | *Formyl peptide receptor 1* |
|  | x | - | 0.397 | *Rtp4* | *Receptor transporter protein 4* |
|  | x | - | 0.381 | *Pde4b* | *phosphodiesterase 4B, cAMP-specific* |
|  | x | - | 0.380 | *Kcna3* | *Potassium voltage-gated channel, shaker-related subfamily, member 3* |
|  | x | - | 0.380 | *Lck* | *Lymphocyte-specific protein tyrosine kinase* |
|  | x | - | 0.363 | *Ccl17* | *Chemokine (C-C motif) ligand 17* |
|  | x | - | 0.359 | *Ahr* | *Aryl hydrocarbon receptor* |
|  | x | - | 0.339 | *Btg2* | *B cell translocation gene 2, anti-proliferative* |
|  | x | - | 0.337 | *Irf7* | *Interferon regulatory factor 7* |
|  | x | - | 0.333 | *Tacr3* | *Tachykinin receptor 3* |
|  | x | - | 0.304 | *Pdpn* | *Podoplanin* |
|  | x | - | 0.295 | *Eif2ak2* | *Eukaryotic translation initiation factor 2-alpha kinase 2* |
|  | x | - | 0.292 | *Hrh1* | *Histamine receptor H1* |
|  | x | - | 0.290 | *Cd69* | *CD69 antigen* |
|  | x | - | 0.275 | *Mefv* | *Mediterranean fever* |

^A^ Obese gonadal white adipose tissue; ^B^ Atherosclerostic aortae; dysregulated genes are sorted by descending rank metric score in AT or AO.

**Additional file 1. Table S3. Non-common genes from obese AT and atherosclerotic AO** involved in the oxidative phosphorylation pathway.

| **Tissue** | | **Rank metric score** | | **Gene symbol** | **Gene name** |
| --- | --- | --- | --- | --- | --- |
| **AT**^A^ | **AO**^B^ | **AT**^A^ | **AO**^B^ |
| x |  | -1.8620 | - | *Por* | *P450 (cytochrome) oxidoreductase* |
| x |  | -1.6540 | - | *Cyc1* | *Cytochrome c-1* |
| x |  | -1.6040 | - | *Retsat* | *Retinol saturase (all-trans-retinol 13,14-reductase)* |
| x |  | -1.4780 | - | *Ndufs2* | *NADH dehydrogenase (ubiquinone) Fe-S protein 2* |
| x |  | -1.3150 | - | *Uqcrc1* | *Ubiquinol-cytochrome c reductase core protein 1* |
| x |  | -1.2790 | - | *Ndufa3* | *NADH dehydrogenase (ubiquinone) 1 alpha subcomplex, 3* |
| x |  | -1.1560 | - | *Uqcrc* | *Ubiquinol-cytochrome c reductase, complex III subunit VII* |
| x |  | -1.1350 | - | *Ndufs3* | *NADH dehydrogenase (ubiquinone) Fe-S protein 3* |
| x |  | -1.1320 | - | *Ech1* | *Enoyl Coenzyme A hydratase 1, peroxisomal* |
| x |  | -1.0850 | - | *Ndufa7* | *NADH dehydrogenase (ubiquinone) 1 alpha subcomplex, 7 (B14.5a)* |
| x |  | -1.0800 | - | *Idh2* | *Isocitrate dehydrogenase 2 (NADP+), mitochondrial* |
| x |  | -0.9760 | - | *Atp5e* | *ATP synthase, H+ transporting, mitochondrial F1 complex, epsilon subunit* |
| x |  | -0.9730 | - | *Ndufa2* | *NADH dehydrogenase (ubiquinone) 1 alpha subcomplex, 2* |
| x |  | -0.9150 | - | *Ndufb7* | *NADH dehydrogenase (ubiquinone) 1 beta subcomplex, 7* |
| x |  | -0.9090 | - | *Atp5b* | *ATP synthase, H+ transporting mitochondrial F1 complex, beta subunit* |
| x |  | -0.8980 | - | *Cox7a2* | *Cytochrome c oxidase subunit VIIa 2* |
| x |  | -0.8920 | - | *Rhot2* | *Ras homolog gene family, member T2* |
| x |  | -0.8800 | - | *Sdhb* | *Succinate dehydrogenase complex, subunit B, iron sulfur (Ip)* |
| x |  | -0.8470 | - | *Polr2f* | *Polymerase (RNA) II (DNA directed) polypeptide F* |
| x |  | -0.8230 | - | *Etfa* | *Electron transferring flavoprotein, alpha polypeptide* |
| x |  | -0.7900 | - | *Atp5g2* | *ATP synthase, H+ transporting, mitochondrial F0 complex, subunit C2* |
| x |  | -0.7800 | - | *Atp5c1* | *ATP synthase, H+ transporting, mitochondrial F1 complex, gamma polypeptide 1* |
| x |  | -0.7420 | - | *Timm13* | *Translocase of inner mitochondrial membrane 13* |
| x |  | -0.7230 | - | *Surf1* | *Surfeit gene 1* |
| x |  | -0.7130 | - | *Timm8b* | *Translocase of inner mitochondrial membrane 8B* |
| x |  | -0.7090 | - | *Cox17* | *Cytochrome c oxidase assembly protein 17* |
| x |  | -0.6990 | - | *Atp5a1* | *ATP synthase, H+ transporting, mitochondrial F1 complex, alpha subunit 1* |
| x |  | -0.6660 | - | *Mrps12* | *Mitochondrial ribosomal protein S12* |
| x |  | -0.6400 | - | *Decr1* | *2,4-dienoyl CoA reductase 1, mitochondrial* |
| x |  | -0.5750 | - | *Timm17a* | *Translocase of inner mitochondrial membrane 17a* |
|  | x | - | -0.6720 | *Hadhb* | *Hydroxyacyl-CoA dehydrogenase , beta subunit* |
|  | x | - | -0.5320 | *Pdk4* | *Pyruvate dehydrogenase kinase, isozyme 4* |
|  | x | - | -0.5100 | *Dlat* | *Dihydrolipoamide S-acetyltransferase* |
|  | x | - | -0.4550 | *Atp5j2* | *ATP synthase, H+ transporting, mitochondrial F0 complex, subunit F2* |
|  | x | - | -0.4250 | *Slc25a20* | *Solute carrier family 25 (carnitine/acylcarnitine translocase), member 20* |
|  | x | - | -0.4100 | *Acadm* | *Acyl-Coenzyme A dehydrogenase, medium chain* |
|  | x | - | -0.3920 | *Bdh2* | *3-hydroxybutyrate dehydrogenase, type 2* |
|  | x | - | -0.3260 | *Afg3l2* | *AFG3-like AAA ATPase 2* |
|  | x | - | -0.2970 | *Cox15* | *COX15 homolog, cytochrome c oxidase assembly protein 15* |
|  | x | - | -0.2870 | *Ndufc2* | *NADH dehydrogenase (ubiquinone) 1, subcomplex unknown, 2* |
|  | x | - | -0.2770 | *Atp6v1g1* | *ATPase, H+ transporting, lysosomal V1 subunit G1* |
|  | x | - | -0.2770 | *Mtx2* | *Metaxin 2* |
|  | x | - | -0.2450 | *Fxn* | *Frataxin* |
|  | x | - | -0.2370 | *Cycs* | *Cytochrome c, somatic* |
|  | x | - | -0.2370 | *Ndufa6* | *NADH dehydrogenase (ubiquinone) 1 alpha subcomplex, 6 (B14)* |
|  | x | - | -0.2170 | *Supv3l1* | *Suppressor of var1, 3-like 1 (S. cerevisiae)* |
|  | x | - | -0.2090 | *Slc25a5* | *Solute carrier family 25 (mitochondrial carrier, adenine nucleotide translocator), member 5* |
|  | x | - | -0.1880 | *Timm9* | *Translocase of inner mitochondrial membrane 9 homolog* |
|  | x | - | -0.1850 | *Opa1* | *Optic atrophy 1* |
|  | x | - | -0.1780 | *Ndufs1* | *NADH dehydrogenase (ubiquinone) Fe-S protein 1* |
|  | x | - | -0.1760 | *Tomm22* | *Translocase of outer mitochondrial membrane 22 homolog (yeast)* |
|  | x | - | -0.1720 | *Cox7a2l* | *Cytochrome c oxidase subunit VIIa polypeptide 2 like* |
|  | x | - | -0.1620 | *Cox10* | *Cytochrome c oxidase assembly protein 10* |

^A^ Obese gonadal white adipose tissue; ^B^ Atherosclerostic aortae; dysregulated genes are sorted by descending rank metric score in AT or AO.

**Additional file 1. Table S4. Common genes from obese AT and AO involved in the dysregulated pathways from Hallmark gene set.**

| **a) E2F targets** | | | |
| --- | --- | --- | --- |
| **Gene symbol** | **Gene name** | **Rank metric score** | |
|  |  | **AT^A^** | **AO^B^** |
| *Mki67* | *Antigen Identified By Monoclonal Antibody Ki-67* | 2.86 | 1.72 |
| *Top2a* | *Topoisomerase (Dna) Ii Alpha 170kda* | 2.66 | 1.29 |
| *Hmmr* | *Hyaluronan-Mediated Motility Receptor (Rhamm)* | 2.44 | 0.24 |
| *Pcna* | *Proliferating Cell Nuclear Antigen* | 2.38 | 0.39 |
| *Cenpe* | *Centromere Protein E, 312kda* | 2.32 | 0.78 |
| *Ccnb2* | *Cyclin B2* | 2.25 | 0.40 |
| *Brca1* | *Breast Cancer 1, Early Onset* | 2.08 | 0.26 |
| *Cks2* | *Cdc28 Protein Kinase Regulatory Subunit 2* | 2.08 | 0.79 |
| *Atad2* | *Atpase Family, Aaa Domain Containing 2* | 2.03 | 0.28 |
| *Mcm3* | *Mcm3 Minichromosome Maintenance Deficient 3 (S. Cerevisiae)* | 1.83 | 0.31 |
| *Tacc3* | *Transforming, Acidic Coiled-Coil Containing Protein 3* | 1.78 | 0.50 |
| *Smc4* | *Structural Maintenance Of Chromosomes 4* | 1.71 | 0.39 |
| *Bub1b* | *Bub1 Budding Uninhibited By Benzimidazoles 1 Homolog Beta (Yeast)* | 1.64 | 0.26 |
| *E2f8* | *E2f Transcription Factor 8* | 1.63 | 0.72 |
| *Asf1b* | *Asf1 Anti-Silencing Function 1 Homolog B (S. Cerevisiae)* | 1.60 | 0.48 |
| *Cdkn3* | *Cyclin-Dependent Kinase Inhibitor 3 (Cdk2-Associated Dual Specificity Phosphatase)* | 1.28 | 0.31 |
| *Plk4* | *Polo-Like Kinase 4 (Drosophila)* | 1.18 | 0.59 |
| *Aurkb* | *Aurora Kinase B* | 1.05 | 0.51 |
| *Lmnb1* | *Lamin B1* | 1.04 | 0.26 |
| *Ezh2* | *Enhancer Of Zeste Homolog 2 (Drosophila)* | 1.01 | 0.46 |
| *Plk1* | *Polo-Like Kinase 1 (Drosophila)* | 0.92 | 0.24 |
| *Aurka* | *Aurora Kinase A* | 0.86 | 0.25 |
| *Syncrip* | *Synaptotagmin Binding, Cytoplasmic Rna Interacting Protein* | 0.77 | 0.28 |
| *Gins3* | *Gins Complex Subunit 3 (Psf3 Homolog)* | 0.73 | 0.25 |
| *Psip1* | *Pc4 And Sfrs1 Interacting Protein 1* | 0.62 | 0.24 |
| *Ncapd2* | *Non-Smc Condensin I Complex, Subunit D2* | 0.58 | 0.28 |
| *Chek2* | *Chk2 Checkpoint Homolog (S. Pombe)* | 0.57 | 0.94 |
| *Rfc2* | *Replication Factor C (Activator 1) 2, 40kda* | 0.57 | 0.46 |
| *Ssrp1* | *Structure Specific Recognition Protein 1* | 0.55 | 0.25 |
| *Hn1* | *Hematological And Neurological Expressed 1* | 0.50 | 0.38 |
| *Tfrc* | *Transferrin Receptor (P90, Cd71)* | 0.42 | 0.59 |
| **b) G2M checkpoint** | | | |
| **Gene symbol** | **Gene name** | **Rank metric score** | |
|  |  | **AT^A^** | **AO^B^** |
| *Mki67* | *Antigen Identified By Monoclonal Antibody Ki-67* | 2.87 | 1.72 |
| *Top2a* | *Topoisomerase (Dna) Ii Alpha 170kda* | 2.66 | 1.29 |
| *Tpx2* | *Tpx2, Microtubule-Associated, Homolog (Xenopus Laevis)* | 2.61 | 1.20 |
| *Kif11* | *Kinesin Family Member 11* | 2.55 | 0.89 |
| *Bub1* | *Bub1 Budding Uninhibited By Benzimidazoles 1 Homolog (Yeast)* | 2.54 | 0.23 |
| *Ccna2* | *Cyclin A2* | 2.49 | 1.12 |
| *Hmmr* | *Hyaluronan-Mediated Motility Receptor (Rhamm)* | 2.44 | 0.24 |
| *Cenpe* | *Centromere Protein E, 312kda* | 2.32 | 0.78 |
| *Mad2l1* | *Mad2 Mitotic Arrest Deficient-Like 1 (Yeast)* | 2.32 | 0.22 |
| *Ccnb2* | *Cyclin B2* | 2.25 | 0.40 |
| *Sap30* | *Sin3a-Associated Protein, 30kda* | 2.15 | 0.52 |
| *Stmn1* | *Stathmin 1/Oncoprotein 18* | 2.14 | 0.19 |
| *Cks2* | *Cdc28 Protein Kinase Regulatory Subunit 2* | 2.08 | 0.79 |
| *Casc5* | *Cancer Susceptibility Candidate 5* | 2.05 | 0.48 |
| *Dbf4* | *Dbf4 Homolog (S. Cerevisiae)* | 2.01 | 0.53 |
| *Ttk* | *Ttk Protein Kinase* | 1.98 | 0.23 |
| *Kif23* | *Kinesin Family Member 23* | 1.97 | 0.58 |
| *Nusap1* | *Nucleolar And Spindle Associated Protein 1* | 1.95 | 0.76 |
| *Smc2* | *Structural Maintenance Of Chromosomes 2* | 1.94 | 0.32 |
| *Kif15* | *Kinesin Family Member 15* | 1.93 | 0.19 |
| *Incenp* | *Inner Centromere Protein Antigens 135/155kda* | 1.88 | 0.18 |
| *Mcm3* | *Mcm3 Minichromosome Maintenance Deficient 3 (S. Cerevisiae)* | 1.83 | 0.31 |
| *Tacc3* | *Transforming, Acidic Coiled-Coil Containing Protein 3* | 1.78 | 0.50 |
| *Smc4* | *Structural Maintenance Of Chromosomes 4* | 1.71 | 0.39 |
| *Tgfb1* | *Transforming Growth Factor, Beta 1 (Camurati-Engelmann Disease)* | 1.65 | 0.52 |
| *Pbk* | *Pdz Binding Kinase* | 1.63 | 0.25 |
| *Exo1* | *Exonuclease 1* | 1.35 | 0.42 |
| *Cdkn3* | *Cyclin-Dependent Kinase Inhibitor 3 (Cdk2-Associated Dual Specificity Phosphatase)* | 1.28 | 0.31 |
| *Polq* | *Polymerase (Dna Directed), Theta* | 1.25 | 0.18 |
| *Amd1* | *Adenosylmethionine Decarboxylase 1* | 1.21 | 0.19 |
| *Plk4* | *Polo-Like Kinase 4 (Drosophila)* | 1.18 | 0.59 |
| *Ccnd1* | *Cyclin D1* | 1.13 | 0.43 |
| *Aurkb* | *Aurora Kinase B* | 1.05 | 0.51 |
| *Lmnb1* | *Lamin B1* | 1.04 | 0.26 |
| *Ezh2* | *Enhancer Of Zeste Homolog 2 (Drosophila)* | 1.01 | 0.46 |
| *Whsc1* | *Wolf-Hirschhorn Syndrome Candidate 1* | 0.97 | 0.19 |
| *Plk1* | *Polo-Like Kinase 1 (Drosophila)* | 0.92 | 0.24 |
| *Aurka* | *Aurora Kinase A* | 0.86 | 0.25 |
| *Cdc7* | *Cdc7 Cell Division Cycle 7 (S. Cerevisiae)* | 0.85 | 0.51 |
| *Syncrip* | *Synaptotagmin Binding, Cytoplasmic Rna Interacting Protein* | 0.77 | 0.28 |
| *Nup50* | *Nucleoporin 50kda* | 0.75 | 0.28 |
| *Troap* | *Trophinin Associated Protein (Tastin)* | 0.60 | 0.34 |
| *E2f1* | *E2f Transcription Factor 1* | 0.57 | 0.44 |
| *Hn1* | *Hematological And Neurological Expressed 1* | 0.49 | 0.38 |
| *Ctcf* | *Ccctc-Binding Factor (Zinc Finger Protein)* | 0.48 | 0.19 |
| **c) Allograft rejection** | | | |
| **Gene symbol** | **Gene name** | **Rank metric score** | |
|  |  | **AT^A^** | **AO^B^** |
| *Igsf6* | *Immunoglobulin Superfamily, Member 6* | 3.77 | 1.89 |
| *Ctss* | *Cathepsin S* | 3.75 | 2.23 |
| *Ccr5* | *Chemokine (C-C Motif) Receptor 5* | 3.64 | 0.75 |
| *Tlr1* | *Toll-Like Receptor 1* | 3.49 | 0.58 |
| *Itgb2* | *Integrin, Beta 2 (Complement Component 3 Receptor 3 And 4 Subunit)* | 3.16 | 0.79 |
| *Tpd52* | *Tumor Protein D52* | 3.03 | 0.31 |
| *Ly86* | *Lymphocyte Antigen 86* | 2.95 | 1.28 |
| *Ptprc* | *Protein Tyrosine Phosphatase, Receptor Type, C* | 2.94 | 1.22 |
| *Ptpn6* | *Protein Tyrosine Phosphatase, Non-Receptor Type 6* | 2.85 | 0.81 |
| *Capg* | *Capping Protein (Actin Filament), Gelsolin-Like* | 2.73 | 0.68 |
| *Gpr65* | *G Protein-Coupled Receptor 65* | 2.53 | 0.91 |
| *Timp1* | *Timp Metallopeptidase Inhibitor 1* | 2.35 | 1.91 |
| *Hcls1* | *Hematopoietic Cell-Specific Lyn Substrate 1* | 2.29 | 0.41 |
| *St8sia4* | *St8 Alpha-N-Acetyl-Neuraminide Alpha-2,8-Sialyltransferase 4* | 2.19 | 0.76 |
| *Was* | *Wiskott-Aldrich Syndrome (Eczema-Thrombocytopenia)* | 2.18 | 0.90 |
| *Ncf4* | *Neutrophil Cytosolic Factor 4, 40kda* | 2.10 | 0.49 |
| *Tgfb1* | *Transforming Growth Factor, Beta 1 (Camurati-Engelmann Disease)* | 1.65 | 0.52 |
| *Ccl2* | *Chemokine (C-C Motif) Ligand 2* | 1.45 | 0.92 |
| *Map4k1* | *Mitogen-Activated Protein Kinase Kinase Kinase Kinase 1* | 1.40 | 0.41 |
| *Fgr* | *Gardner-Rasheed Feline Sarcoma Viral (V-Fgr) Oncogene Homolog* | 1.32 | 0.82 |
| *Bcat1* | *Branched Chain Aminotransferase 1, Cytosolic* | 1.19 | 0.28 |
| *Ccl7* | *Chemokine (C-C Motif) Ligand 7* | 1.13 | 0.96 |
| *Fcgr2b* | *Fc Fragment Of Igg, Low Affinity Iib, Receptor (Cd32)* | 0.99 | 0.78 |
| *Ccl5* | *Chemokine (C-C Motif) Ligand 5* | 0.90 | 0.70 |
| *Icam1* | *Intercellular Adhesion Molecule 1 (Cd54), Human Rhinovirus Receptor* | 0.88 | 0.78 |
| *Ccr2* | *Chemokine (C-C Motif) Receptor 2* | 0.85 | 0.37 |
| *Cd47* | *Cd47 Molecule* | 0.81 | 0.39 |
| *Elf4* | *E74-Like Factor 4 (Ets Domain Transcription Factor)* | 0.81 | 0.29 |
| **d) IL6 JAK STAT3 signaling** | | | |
| **Gene symbol** | **Gene name** | **Rank metric score** | |
|  |  | **AT^A^** | **AO^B^** |
| *Cd44* | *Cd44 Molecule (Indian Blood Group)* | 2.99 | 0.37 |
| *Pik3r5* | *Phosphoinositide-3-Kinase, Regulatory Subunit 5, P101* | 2.96 | 1.32 |
| *Hmox1* | *Heme Oxygenase (Decycling) 1* | 2.29 | 1.79 |
| *Il13ra1* | *Interleukin 13 Receptor, Alpha 1* | 1.99 | 0.72 |
| *Cd14* | *Cd14 Molecule* | 1.91 | 0.25 |
| *Il10rb* | *Interleukin 10 Receptor, Beta* | 1.84 | 0.71 |
| *Csf2ra* | *Colony Stimulating Factor 2 Receptor, Alpha, Low-Affinity (Granulocyte-Macrophage)* | 1.69 | 0.30 |
| *Tgfb1* | *Transforming Growth Factor, Beta 1 (Camurati-Engelmann Disease)* | 1.65 | 0.52 |
| *Ptpn1* | *Protein Tyrosine Phosphatase, Non-Receptor Type 1* | 1.44 | 0.48 |
| *Ifnar1* | *Interferon (Alpha, Beta And Omega) Receptor 1* | 1.30 | 0.27 |
| *Pf4* | *Platelet Factor 4 (Chemokine (C-X-C Motif) Ligand 4)* | 1.20 | 0.26 |
| *Map3k8* | *Mitogen-Activated Protein Kinase Kinase Kinase 8* | 1.16 | 0.32 |
| *Ccl7* | *Chemokine (C-C Motif) Ligand 7* | 1.13 | 0.96 |
| *Csf3r* | *Colony Stimulating Factor 3 Receptor (Granulocyte)* | 1.09 | 1.06 |
| *Il1r2* | *Interleukin 1 Receptor, Type Ii* | 0.95 | 0.25 |
| *Il1r1* | *Interleukin 1 Receptor, Type I* | 0.92 | 0.46 |
| *Crlf2* | *Cytokine Receptor-Like Factor 2* | 0.62 | 0.26 |
| *Osmr* | *Oncostatin M Receptor* | 0.60 | 0.61 |
| *Cxcl1* | *Chemokine (C-X-C Motif) Ligand 1 (Melanoma Growth Stimulating Activity, Alpha)* | 0.59 | 0.50 |
| **e) Epithelial mesenchymal transition** | | | |
| **Gene symbol** | **Gene name** | **Rank metric score** | |
|  |  | **AT^A^** | **AO^B^** |
| *Glipr1* | *Gli Pathogenesis-Related 1 (Glioma)* | 4.25 | 0.36 |
| *Anpep* | *Alanyl (Membrane) Aminopeptidase* | 3.55 | 1.20 |
| *Lum* | *Lumican* | 3.21 | 0.91 |
| *Cd44* | *Cd44 Molecule (Indian Blood Group)* | 2.99 | 0.37 |
| *Mmp2* | *Matrix Metallopeptidase 2 (Gelatinase A, 72kda Gelatinase, 72kda Type Iv Collagenase)* | 2.98 | 0.43 |
| *Capg* | *Capping Protein (Actin Filament), Gelsolin-Like* | 2.73 | 0.68 |
| *Mmp3* | *Matrix Metallopeptidase 3 (Stromelysin 1, Progelatinase)* | 2.49 | 0.66 |
| *Mgp* | *Matrix Gla Protein* | 2.38 | 0.51 |
| *Timp1* | *Timp Metallopeptidase Inhibitor 1* | 2.35 | 1.91 |
| *Plod2* | *Procollagen-Lysine, 2-Oxoglutarate 5-Dioxygenase 2* | 1.91 | 0.30 |
| *Sat1* | *Spermidine/Spermine N1-Acetyltransferase 1* | 1.87 | 0.43 |
| *Col12a1* | *Collagen, Type Xii, Alpha 1* | 1.79 | 0.36 |
| *Plaur* | *Plasminogen Activator, Urokinase Receptor* | 1.69 | 0.52 |
| *Tgfb1* | *Transforming Growth Factor, Beta 1 (Camurati-Engelmann Disease)* | 1.65 | 0.52 |
| *Vcam1* | *Vascular Cell Adhesion Molecule 1* | 1.58 | 1.50 |
| *Col6a3* | *Collagen, Type Vi, Alpha 3* | 1.31 | 0.31 |
| *Lrp1* | *Low Density Lipoprotein-Related Protein 1 (Alpha-2-Macroglobulin Receptor)* | 1.25 | 0.33 |
| *Sdc1* | *Syndecan 1* | 1.22 | 0.44 |
| *Lox* | *Lysyl Oxidase* | 1.17 | 0.58 |
| *Pcolce* | *Procollagen C-Endopeptidase Enhancer* | 1.16 | 0.24 |
| *Col5a2* | *Collagen, Type V, Alpha 2* | 0.95 | 0.41 |
| *Gadd45a* | *Growth Arrest And Dna-Damage-Inducible, Alpha* | 0.95 | 1.01 |
| *Tnfaip3* | *Tumor Necrosis Factor, Alpha-Induced Protein 3* | 0.78 | 0.73 |
| *Mest* | *Mesoderm Specific Transcript Homolog (Mouse)* | 0.66 | 0.98 |
| *Sfrp4* | *Secreted Frizzled-Related Protein 4* | 0.63 | 0.45 |
| *Cdh11* | *Cadherin 11, Type 2, Ob-Cadherin (Osteoblast)* | 0.61 | 0.33 |
| *Cxcl1* | *Chemokine (C-X-C Motif) Ligand 1 (Melanoma Growth Stimulating Activity, Alpha)* | 0.59 | 0.50 |
| *Crlf1* | *Cytokine Receptor-Like Factor 1* | 0.57 | 1.26 |
| *Sfrp1* | *Secreted Frizzled-Related Protein 1* | 0.57 | 0.24 |
| **f) TNFA signaling via NFKB** | | | |
| **Gene symbol** | **Gene name** | **Rank metric score** | |
|  |  | **AT^A^** | **AO^B^** |
| *Il7r* | *Interleukin 7 Receptor* | 4.55 | 2.02 |
| *Atf3* | *Activating Transcription Factor 3* | 3.61 | 0.62 |
| *Plek* | *Pleckstrin* | 3.39 | 1.27 |
| *B4galt5* | *Udp-Gal:Betaglcnac Beta 1,4- Galactosyltransferase, Polypeptide 5* | 3.01 | 0.72 |
| *Cd44* | *Cd44 Molecule (Indian Blood Group)* | 2.99 | 0.37 |
| *Plk2* | *Polo-Like Kinase 2 (Drosophila)* | 2.90 | 0.92 |
| *Tnfaip2* | *Tumor Necrosis Factor, Alpha-Induced Protein 2* | 2.58 | 0.91 |
| *Ptpre* | *Protein Tyrosine Phosphatase, Receptor Type, E* | 2.42 | 0.89 |
| *Rel* | *V-Rel Reticuloendotheliosis Viral Oncogene Homolog (Avian)* | 2.30 | 0.60 |
| *Plau* | *Plasminogen Activator, Urokinase* | 2.05 | 0.61 |
| *Sat1* | *Spermidine/Spermine N1-Acetyltransferase 1* | 1.86 | 0.43 |
| *Panx1* | *Pannexin 1* | 1.75 | 0.33 |
| *Plaur* | *Plasminogen Activator, Urokinase Receptor* | 1.69 | 0.52 |
| *Fos* | *V-Fos Fbj Murine Osteosarcoma Viral Oncogene Homolog* | 1.64 | 0.36 |
| *Ccl2* | *Chemokine (C-C Motif) Ligand 2* | 1.45 | 0.92 |
| *Map3k8* | *Mitogen-Activated Protein Kinase Kinase Kinase 8* | 1.16 | 0.32 |
| *Ccnd1* | *Cyclin D1* | 1.13 | 0.43 |
| *Tubb2a* | *Tubulin, Beta 2a* | 1.02 | 0.85 |
| *Gadd45a* | *Growth Arrest And Dna-Damage-Inducible, Alpha* | 0.95 | 1.01 |
| *Ccl5* | *Chemokine (C-C Motif) Ligand 5* | 0.90 | 0.70 |
| *Icam1* | *Intercellular Adhesion Molecule 1 (Cd54), Human Rhinovirus Receptor* | 0.88 | 0.78 |
| *Tnfaip3* | *Tumor Necrosis Factor, Alpha-Induced Protein 3* | 0.78 | 0.73 |
| *Cxcl2* | *Chemokine (C-X-C Motif) Ligand 2* | 0.73 | 0.74 |
| *Cxcl1* | *Chemokine (C-X-C Motif) Ligand 1 (Melanoma Growth Stimulating Activity, Alpha)* | 0.59 | 0.50 |
| *Abca1* | *Atp-Binding Cassette, Sub-Family A (Abc1), Member 1* | 0.57 | 0.99 |
| *Inhba* | *Inhibin, Beta A (Activin A, Activin Ab Alpha Polypeptide)* | 0.49 | 0.63 |
| *Nfkb2* | *Nuclear Factor Of Kappa Light Polypeptide Gene Enhancer In B-Cells 2 (P49/P100)* | 0.49 | 0.44 |
| *Dusp1* | *Dual Specificity Phosphatase 1* | 0.45 | 0.70 |
| **g) Mitotic spindle** | | | |
| **Gene symbol** | **Gene name** | **Rank metric score** | |
|  |  | **AT^A^** | **AO^B^** |
| *Dock2* | *Dedicator Of Cytokinesis 2* | 3.19 | 1.05 |
| *Rasa1* | *Ras P21 Protein Activator (Gtpase Activating Protein) 1* | 2.71 | 0.18 |
| *Top2a* | *Topoisomerase (Dna) Ii Alpha 170kda* | 2.66 | 1.29 |
| *Tpx2* | *Tpx2, Microtubule-Associated, Homolog (Xenopus Laevis)* | 2.61 | 1.20 |
| *Kif11* | *Kinesin Family Member 11* | 2.55 | 0.89 |
| *Bub1* | *Bub1 Budding Uninhibited By Benzimidazoles 1 Homolog (Yeast)* | 2.54 | 0.23 |
| *Ect2* | *Epithelial Cell Transforming Sequence 2 Oncogene* | 2.49 | 0.84 |
| *Cenpe* | *Centromere Protein E, 312kda* | 2.32 | 0.78 |
| *Ccnb2* | *Cyclin B2* | 2.25 | 0.40 |
| *Ttk* | *Ttk Protein Kinase* | 1.98 | 0.23 |
| *Kif23* | *Kinesin Family Member 23* | 1.97 | 0.58 |
| *Nusap1* | *Nucleolar And Spindle Associated Protein 1* | 1.95 | 0.76 |
| *Kif15* | *Kinesin Family Member 15* | 1.93 | 0.19 |
| *Incenp* | *Inner Centromere Protein Antigens 135/155kda* | 1.88 | 0.18 |
| *Smc4* | *Structural Maintenance Of Chromosomes 4* | 1.71 | 0.39 |
| *Prex1* | *-* | 1.56 | 0.41 |
| *Arf6* | *Adp-Ribosylation Factor 6* | 1.42 | 0.41 |
| *Abi1* | *Abl-Interactor 1* | 1.29 | 0.19 |
| *Lmnb1* | *Lamin B1* | 1.04 | 0.26 |
| *Plk1* | *Polo-Like Kinase 1 (Drosophila)* | 0.92 | 0.24 |
| *Aurka* | *Aurora Kinase A* | 0.86 | 0.25 |
| *Nin* | *Ninein (Gsk3b Interacting Protein)* | 0.84 | 0.52 |
| **h) Complement** | | | |
| **Gene symbol** | **Gene name** | **Rank metric score** | |
|  |  | **AT^A^** | **AO^B^** |
| *Ctss* | *Cathepsin S* | 3.75 | 2.24 |
| *Plek* | *Pleckstrin* | 3.39 | 1.27 |
| *Lgmn* | *Legumain* | 3.14 | 1.43 |
| *Pla2g7* | *Phospholipase A2, Group Vii (Platelet-Activating Factor Acetylhydrolase, Plasma)* | 3.14 | 0.96 |
| *Itgam* | *Integrin, Alpha M (Complement Component 3 Receptor 3 Subunit)* | 3.01 | 0.77 |
| *Casp1* | *Caspase 1, Apoptosis-Related Cysteine Peptidase (Interleukin 1, Beta, Convertase)* | 2.99 | 0.60 |
| *Lgals3* | *Lectin, Galactoside-Binding, Soluble, 3 (Galectin 3)* | 2.89 | 2.61 |
| *Fcer1g* | *Fc Fragment Of Ige, High Affinity I, Receptor For; Gamma Polypeptide* | 2.68 | 1.40 |
| *Timp1* | *Timp Metallopeptidase Inhibitor 1* | 2.35 | 1.91 |
| *C1qc* | *Complement Component 1, Q Subcomponent, C Chain* | 2.30 | 1.34 |
| *Was* | *Wiskott-Aldrich Syndrome (Eczema-Thrombocytopenia)* | 2.18 | 0.90 |
| *C1qa* | *Complement Component 1, Q Subcomponent, A Chain* | 2.04 | 0.92 |
| *Ctsh* | *Cathepsin H* | 1.97 | 0.52 |
| *Mmp13* | *Matrix Metallopeptidase 13 (Collagenase 3)* | 1.72 | 0.44 |
| *Plaur* | *Plasminogen Activator, Urokinase Receptor* | 1.69 | 0.52 |
| *Lrp1* | *Low Density Lipoprotein-Related Protein 1 (Alpha-2-Macroglobulin Receptor)* | 1.25 | 0.33 |
| *Ctsb* | *Cathepsin B* | 1.05 | 0.77 |
| *Ctsd* | *Cathepsin D (Lysosomal Aspartyl Peptidase)* | 1.03 | 0.76 |
| *Ccl5* | *Chemokine (C-C Motif) Ligand 5* | 0.90 | 0.68 |
| *Tnfaip3* | *Tumor Necrosis Factor, Alpha-Induced Protein 3* | 0.78 | 0.73 |
| *Cxcl1* | *Chemokine (C-X-C Motif) Ligand 1 (Melanoma Growth Stimulating Activity, Alpha)* | 0.59 | 0.50 |
| *Gngt2* | *Guanine Nucleotide Binding Protein, Gamma Transducing Activity Polypeptide 2* | 0.58 | 0.43 |
| *Gnai2* | *Guanine Nucleotide Binding Protein, Alpha Inhibiting Activity Polypeptide 2* | 0.54 | 0.37 |
| **i) Kras signaling up** | | | |
| **Gene symbol** | **Gene name** | **Rank metric score** | |
|  |  | **AT^A^** | **AO^B^** |
| *Gpnmb* | *Glycoprotein (Transmembrane) Nmb* | 4.74 | 2.00 |
| *Il7r* | *Interleukin 7 Receptor* | 4.55 | 2.02 |
| *Adam8* | *Adam Metallopeptidase Domain 8* | 4.40 | 1.68 |
| *Ctss* | *Cathepsin S* | 3.74 | 2.24 |
| *C3ar1* | *Complement Component 3a Receptor 1* | 3.70 | 1.62 |
| *Tlr8* | *Toll-Like Receptor 8* | 3.69 | 1.45 |
| *Lat2* | *Linker For Activation Of T Cells Family, Member 2* | 3.62 | 0.48 |
| *Lcp1* | *Lymphocyte Cytosolic Protein 1 (L-Plastin)* | 3.25 | 1.55 |
| *Dock2* | *Dedicator Of Cytokinesis 2* | 3.19 | 1.06 |
| *Itgb2* | *Integrin, Beta 2 (Complement Component 3 Receptor 3 And 4 Subunit)* | 3.16 | 0.79 |
| *Fcer1g* | *Fc Fragment Of Ige, High Affinity I, Receptor For; Gamma Polypeptide* | 2.68 | 1.40 |
| *Laptm5* | *Lysosomal Associated Multispanning Membrane Protein 5* | 2.41 | 1.25 |
| *Il10ra* | *Interleukin 10 Receptor, Alpha* | 2.20 | 1.83 |
| *Mafb* | *V-Maf Musculoaponeurotic Fibrosarcoma Oncogene Homolog B (Avian)* | 2.17 | 0.79 |
| *Cxcr4* | *Chemokine (C-X-C Motif) Receptor 4* | 2.14 | 1.13 |
| *Plau* | *Plasminogen Activator, Urokinase* | 2.05 | 0.61 |
| *Cd37* | *Cd37 Molecule* | 1.86 | 0.70 |
| *Ikzf1* | *Ikaros Family Zinc Finger 1 (Ikaros)* | 1.76 | 1.09 |
| *Plaur* | *Plasminogen Activator, Urokinase Receptor* | 1.69 | 0.52 |
| *Map4k1* | *Mitogen-Activated Protein Kinase Kinase Kinase Kinase 1* | 1.40 | 0.41 |
| *Apod* | *Apolipoprotein D* | 1.39 | 0.52 |
| *Tmem176a* | *Transmembrane Protein 176a* | 0.87 | 0.51 |
| *Nin* | *Ninein (Gsk3b Interacting Protein)* | 0.84 | 0.52 |
| *Tnfaip3* | *Tumor Necrosis Factor, Alpha-Induced Protein 3* | 0.78 | 0.73 |
| **j) Protein secretion** | | | |
| **Gene symbol** | **Gene name** | **Rank metric score** | |
|  |  | **AT^A^** | **AO^B^** |
| *Gla* | *Galactosidase, Alpha* | 3.53 | 0.80 |
| *Tpd52* | *Tumor Protein D52* | 3.03 | 0.31 |
| *Galc* | *Galactosylceramidase* | 2.67 | 0.96 |
| *Atp1a1* | *Atpase, Na+/K+ Transporting, Alpha 1 Polypeptide* | 1.99 | 0.33 |
| *Cd63* | *Cd63 Molecule* | 1.89 | 0.49 |
| *Vamp4* | *Vesicle-Associated Membrane Protein 4* | 1.54 | 0.52 |
| *Stx7* | *Syntaxin 7* | 1.33 | 0.24 |
| *Snx2* | *Sorting Nexin 2* | 0.84 | 0.19 |
| *Rab5a* | *Rab5a, Member Ras Oncogene Family* | 0.59 | 0.18 |
| *Abca1* | *Atp-Binding Cassette, Sub-Family A (Abc1), Member 1* | 0.57 | 0.99 |
| *Clta* | *Clathrin, Light Chain (Lca)* | 0.55 | 0.21 |
| *Ppt1* | *Palmitoyl-Protein Thioesterase 1 (Ceroid-Lipofuscinosis, Neuronal 1, Infantile)* | 0.43 | 0.20 |
| *Atp6v1b1* | *Atpase, H+ Transporting, Lysosomal 56/58kda, V1 Subunit B1* | 0.38 | 0.53 |
| *Ctsc* | *Cathepsin C* | 0.38 | 0.20 |
| *Arfgef2* | *Adp-Ribosylation Factor Guanine Nucleotide-Exchange Factor 2 (Brefeldin A-Inhibited)* | 0.31 | 0.23 |
| *Ap2s1* | *Adaptor-Related Protein Complex 2, Sigma 1 Subunit* | 0.30 | 0.16 |
| **k) Apoptosis** | | | |
| **Gene symbol** | **Gene name** | **Rank metric score** | |
|  |  | **AT^A^** | **AO^B^** |
| *Atf3* | *Activating Transcription Factor 3* | 3.61 | 0.62 |
| *Lum* | *Lumican* | 3.21 | 0.91 |
| *Casp1* | *Caspase 1, Apoptosis-Related Cysteine Peptidase (Interleukin 1, Beta, Convertase)* | 2.99 | 0.60 |
| *Cd44* | *Cd44 Molecule (Indian Blood Group)* | 2.99 | 0.37 |
| *Mmp2* | *Matrix Metallopeptidase 2 (Gelatinase A, 72kda Gelatinase, 72kda Type Iv Collagenase)* | 2.98 | 0.43 |
| *Lgals3* | *Lectin, Galactoside-Binding, Soluble, 3 (Galectin 3)* | 2.90 | 2.61 |
| *Top2a* | *Topoisomerase (Dna) Ii Alpha 170kda* | 2.66 | 1.29 |
| *Timp1* | *Timp Metallopeptidase Inhibitor 1* | 2.35 | 1.91 |
| *Hmox1* | *Heme Oxygenase (Decycling) 1* | 2.29 | 1.79 |
| *Hgf* | *Hepatocyte Growth Factor (Hepapoietin A; Scatter Factor)* | 2.24 | 1.35 |
| *Sat1* | *Spermidine/Spermine N1-Acetyltransferase 1* | 1.87 | 0.43 |
| *Gna15* | *Guanine Nucleotide Binding Protein (G Protein), Alpha 15 (Gq Class)* | 1.54 | 0.36 |
| *Anxa1* | *Annexin A1* | 1.29 | 0.59 |
| *Ccnd1* | *Cyclin D1* | 1.13 | 0.43 |
| *Gadd45a* | *Growth Arrest And Dna-Damage-Inducible, Alpha* | 0.95 | 1.01 |
| *Gsr* | *Glutathione Reductase* | 0.78 | 0.51 |
| *Cd69* | *Cd69 Molecule* | 0.76 | 0.29 |
| *Pmaip1* | *Phorbol-12-Myristate-13-Acetate-Induced Protein 1* | 0.73 | 0.41 |
| **l) Interferon gamma response** | | | |
| **Gene symbol** | **Gene name** | **Rank metric score** | |
|  |  | **AT^A^** | **AO^B^** |
| *Slamf7* | *Slam Family Member 7* | 4.01 | 2.11 |
| *Casp1* | *Caspase 1, Apoptosis-Related Cysteine Peptidase (Interleukin 1, Beta, Convertase)* | 2.99 | 0.60 |
| *Ifi30* | *Interferon, Gamma-Inducible Protein 30* | 2.98 | 0.60 |
| *Ptpn6* | *Protein Tyrosine Phosphatase, Non-Receptor Type 6* | 2.85 | 0.81 |
| *Tnfaip2* | *Tumor Necrosis Factor, Alpha-Induced Protein 2* | 2.58 | 0.91 |
| *Il10ra* | *Interleukin 10 Receptor, Alpha* | 2.20 | 1.83 |
| *P2ry14* | *Purinergic Receptor P2y, G-Protein Coupled, 14* | 2.13 | 0.94 |
| *Vcam1* | *Vascular Cell Adhesion Molecule 1* | 1.58 | 1.50 |
| *Samhd1* | *Sam Domain And Hd Domain 1* | 1.48 | 0.38 |
| *Ccl2* | *Chemokine (C-C Motif) Ligand 2* | 1.45 | 0.92 |
| *Epsti1* | *Epithelial Stromal Interaction 1 (Breast)* | 1.44 | 0.88 |
| *Ptpn1* | *Protein Tyrosine Phosphatase, Non-Receptor Type 1* | 1.44 | 0.48 |
| *Ccl7* | *Chemokine (C-C Motif) Ligand 7* | 1.13 | 0.96 |
| *Trim25* | *Tripartite Motif-Containing 25* | 1.10 | 0.69 |
| *Ccl5* | *Chemokine (C-C Motif) Ligand 5* | 0.90 | 0.70 |
| *Icam1* | *Intercellular Adhesion Molecule 1 (Cd54), Human Rhinovirus Receptor* | 0.88 | 0.78 |
| *Parp14* | *Poly (Adp-Ribose) Polymerase Family, Member 14* | 0.88 | 0.56 |
| *Tnfaip3* | *Tumor Necrosis Factor, Alpha-Induced Protein 3* | 0.78 | 0.73 |
| *Cd69* | *Cd69 Molecule* | 0.76 | 0.29 |
| **m) Angiogenesis** | | | |
| **Gene symbol** | **Gene name** | **Rank metric score** | |
|  |  | **AT^A^** | **AO^B^** |
| *Lum* | *Lumican* | 3.21 | 0.91 |
| *Timp1* | *Timp Metallopeptidase Inhibitor 1* | 2.35 | 1.91 |
| *S100a4* | *S100 Calcium Binding Protein A4* | 1.98 | 1.27 |
| *Col5a2* | *Collagen, Type V, Alpha 2* | 0.95 | 0.41 |
| **n) Coagulation** | | | |
| **Gene symbol** | **Gene name** | **Rank metric score** | |
|  |  | **AT^A^** | **AO^B^** |
| *Ctsk* | *Cathepsin K (Pycnodysostosis)* | 4.31 | 0.56 |
| *Plek* | *Pleckstrin* | 3.39 | 1.27 |
| *Lgmn* | *Legumain* | 3.14 | 1.43 |
| *Mmp2* | *Matrix Metallopeptidase 2 (Gelatinase A, 72kda Gelatinase, 72kda Type Iv Collagenase)* | 2.98 | 0.43 |
| *Mmp3* | *Matrix Metallopeptidase 3 (Stromelysin 1, Progelatinase)* | 2.49 | 0.66 |
| *Timp1* | *Timp Metallopeptidase Inhibitor 1* | 2.35 | 1.91 |
| *Plau* | *Plasminogen Activator, Urokinase* | 2.05 | 0.61 |
| *C1qa* | *Complement Component 1, Q Subcomponent, A Chain* | 2.04 | 0.92 |
| *Ctsh* | *Cathepsin H* | 1.97 | 0.52 |
| *Anxa1* | *Annexin A1* | 1.29 | 0.59 |
| *Lrp1* | *Low Density Lipoprotein-Related Protein 1 (Alpha-2-Macroglobulin Receptor)* | 1.25 | 0.33 |
| *Pf4* | *Platelet Factor 4 (Chemokine (C-X-C Motif) Ligand 4)* | 1.20 | 0.26 |
| *Ctsb* | *Cathepsin B* | 1.05 | 0.77 |
| *Cfi* | *Complement Factor I* | 0.67 | 0.27 |
| *Adam9* | *Adam Metallopeptidase Domain 9 (Meltrin Gamma)* | 0.50 | 0.29 |
| *Plat* | *Plasminogen Activator, Tissue* | 0.46 | 0.44 |
| *Mmp8* | *Matrix Metallopeptidase 8 (Neutrophil Collagenase)* | 0.45 | 0.92 |
| **o) P53** | | | |
| **Gene symbol** | **Gene name** | **Rank metric score** | |
|  |  | **AT^A^** | **AO^B^** |
| *Atf3* | *Activating Transcription Factor 3* | 3.61 | 0.62 |
| *Casp1* | *Caspase 1, Apoptosis-Related Cysteine Peptidase (Interleukin 1, Beta, Convertase)* | 2.99 | 0.60 |
| *Ifi30* | *Interferon, Gamma-Inducible Protein 30* | 2.98 | 0.60 |
| *Plk2* | *Polo-Like Kinase 2 (Drosophila)* | 2.90 | 0.92 |
| *Lrmp* | *Lymphoid-Restricted Membrane Protein* | 2.45 | 0.69 |
| *Ptpre* | *Protein Tyrosine Phosphatase, Receptor Type, E* | 2.42 | 0.89 |
| *Pcna* | *Proliferating Cell Nuclear Antigen* | 2.38 | 0.39 |
| *Hmox1* | *Heme Oxygenase (Decycling) 1* | 2.29 | 1.79 |
| *Plxnb2* | *Plexin B2* | 2.05 | 0.26 |
| *Cyfip2* | *Cytoplasmic Fmr1 Interacting Protein 2* | 1.99 | 0.67 |
| *S100a4* | *S100 Calcium Binding Protein A4* | 1.98 | 1.27 |
| *Sat1* | *Spermidine/Spermine N1-Acetyltransferase 1* | 1.87 | 0.43 |
| *Pitpnc1* | *Phosphatidylinositol Transfer Protein, Cytoplasmic 1* | 1.80 | 0.31 |
| *Tgfb1* | *Transforming Growth Factor, Beta 1 (Camurati-Engelmann Disease)* | 1.65 | 0.52 |
| *Fos* | *V-Fos Fbj Murine Osteosarcoma Viral Oncogene Homolog* | 1.64 | 0.36 |
| *F2r* | *Coagulation Factor Ii (Thrombin) Receptor* | 1.59 | 0.24 |
| *Steap3* | *Steap Family Member 3* | 1.30 | 0.46 |
| *Sdc1* | *Syndecan 1* | 1.22 | 0.44 |
| *Ctsd* | *Cathepsin D (Lysosomal Aspartyl Peptidase)* | 1.03 | 0.76 |
| *Gadd45a* | *Growth Arrest And Dna-Damage-Inducible, Alpha* | 0.95 | 1.01 |
| *Polh* | *Polymerase (Dna Directed), Eta* | 0.85 | 0.45 |
| **p) Peroxisome** | | | |
| **Gene symbol** | **Gene name** | **Rank metric score** | |
|  |  | **AT^A^** | **AO^B^** |
| *Ephx2* | *Epoxide Hydrolase 2, Cytoplasmic* | -1.82 | -0.58 |
| *Cat* | *Catalase* | -1.70 | -0.54 |
| *Gnpat* | *Glyceronephosphate O-Acyltransferase* | -1.58 | -0.46 |
| *Ehhadh* | *Enoyl-Coenzyme A, Hydratase/3-Hydroxyacyl Coenzyme A Dehydrogenase* | -1.53 | -1.71 |
| *Crat* | *Carnitine Acetyltransferase* | -1.40 | -0.38 |
| *Slc25a19* | *Solute Carrier Family 25 (Mitochondrial Deoxynucleotide Carrier), Member 19* | -1.34 | -0.22 |
| *Pex11a* | *Peroxisomal Biogenesis Factor 11a* | -1.17 | -0.36 |
| *Rxrg* | *Retinoid X Receptor, Gamma* | -1.14 | -0.28 |
| *Pex5* | *Peroxisomal Biogenesis Factor 5* | -0.91 | -0.33 |
| *Scp2* | *Sterol Carrier Protein 2* | -0.89 | -0.76 |
| **q) Bile acid metabolism** | | | |
| **Gene symbol** | **Gene name** | **Rank metric score** | |
|  |  | **AT^A^** | **AO^B^** |
| *Pxmp2* | *Peroxisomal Membrane Protein 2, 22kda* | -2.66 | -0.72 |
| *Ephx2* | *Epoxide Hydrolase 2, Cytoplasmic* | -1.82 | -0.58 |
| *Cat* | *Catalase* | -1.70 | -0.54 |
| *Gnpat* | *Glyceronephosphate O-Acyltransferase* | -1.58 | -0.46 |
| *Phyh* | *Phytanoyl-Coa 2-Hydroxylase* | -1.50 | -0.72 |
| *Pex19* | *Peroxisomal Biogenesis Factor 19* | -1.42 | -0.42 |
| *Hacl1* | *2-Hydroxyacyl-Coa Lyase 1* | -1.37 | -0.65 |
| *Pex11a* | *Peroxisomal Biogenesis Factor 11a* | -1.17 | -0.36 |
| *Rxrg* | *Retinoid X Receptor, Gamma* | -1.15 | -0.28 |
| *Ar* | *Androgen Receptor (Dihydrotestosterone Receptor; Testicular Feminization; Spinal And Bulbar Muscular Atrophy; Kennedy Disease)* | -1.12 | -0.34 |
| *Paox* | *Polyamine Oxidase (Exo-N4-Amino)* | -1.05 | -0.37 |
| *Scp2* | *Sterol Carrier Protein 2* | -0.90 | -0.76 |
| *Amacr* | *Alpha-Methylacyl-Coa Racemase* | -0.81 | -0.40 |
| **r) Xenobiotic metabolism** | | | |
| **Gene symbol** | **Gene name** | **Rank metric score** | |
|  |  | **AT^A^** | **AO^B^** |
| *Cyp2e1* | *Cytochrome P450, Family 2, Subfamily E, Polypeptide 1* | -3.70 | -3.10 |
| *Hsd11b1* | *Hydroxysteroid (11-Beta) Dehydrogenase 1* | -2.22 | -0.54 |
| *Mccc2* | *Methylcrotonoyl-Coenzyme A Carboxylase 2 (Beta)* | -2.19 | -0.51 |
| *Ddt* | *D-Dopachrome Tautomerase* | -2.13 | -0.45 |
| *Fah* | *Fumarylacetoacetate Hydrolase (Fumarylacetoacetase)* | -2.10 | -0.70 |
| *Slc1a5* | *Solute Carrier Family 1 (Neutral Amino Acid Transporter), Member 5* | -2.08 | -0.59 |
| *Csad* | *Cysteine Sulfinic Acid Decarboxylase* | -2.04 | -0.58 |
| *Cdo1* | *Cysteine Dioxygenase, Type I* | -1.99 | -0.56 |
| *Fmo1* | *Flavin Containing Monooxygenase 1* | -1.96 | -0.80 |
| *Cat* | *Catalase* | -1.71 | -0.54 |
| *Bphl* | *Biphenyl Hydrolase-Like (Serine Hydrolase; Breast Epithelial Mucin-Associated Antigen)* | -1.50 | -0.37 |
| *Atoh8* | *Atonal Homolog 8 (Drosophila)* | -1.46 | -0.39 |
| *Aco2* | *Aconitase 2, Mitochondrial* | -1.39 | -0.38 |
| *Hacl1* | *2-Hydroxyacyl-Coa Lyase 1* | -1.37 | -0.65 |
| *Pts* | *6-Pyruvoyltetrahydropterin Synthase* | -1.15 | -0.39 |
| *Gstm4* | *Glutathione S-Transferase M4* | -0.92 | -0.72 |
| *Fmo3* | *Flavin Containing Monooxygenase 3* | -0.90 | -0.46 |
| **s) Fatty acid metabolism** | | | |
| **Gene symbol** | **Gene name** | **Rank metric score** | |
|  |  | **AT^A^** | **AO^B^** |
| *Acsm3* | *Acyl-Coa Synthetase Medium-Chain Family Member 3* | -2.99 | -1.16 |
| *Fasn* | *Fatty Acid Synthase* | -2.68 | -1.41 |
| *Gpd2* | *Glycerol-3-Phosphate Dehydrogenase 2 (Mitochondrial)* | -2.00 | -0.49 |
| *Fmo1* | *Flavin Containing Monooxygenase 1* | -1.96 | -0.81 |
| *Gstz1* | *Glutathione Transferase Zeta 1 (Maleylacetoacetate Isomerase)* | -1.96 | -0.82 |
| *Acot2* | *Acyl-Coa Thioesterase 2* | -1.77 | -0.39 |
| *Adipor2* | *Adiponectin Receptor 2* | -1.60 | -0.66 |
| *Inmt* | *Indolethylamine N-Methyltransferase* | -1.58 | -0.36 |
| *Ehhadh* | *Enoyl-Coenzyme A, Hydratase/3-Hydroxyacyl Coenzyme A Dehydrogenase* | -1.53 | -1.70 |
| *Bphl* | *Biphenyl Hydrolase-Like (Serine Hydrolase; Breast Epithelial Mucin-Associated Antigen)* | -1.50 | -0.37 |
| *Crat* | *Carnitine Acetyltransferase* | -1.40 | -0.38 |
| *Aco2* | *Aconitase 2, Mitochondrial* | -1.39 | -0.38 |
| *Alad* | *Aminolevulinate, Delta-, Dehydratase* | -1.33 | -0.30 |
| *Me1* | *Malic Enzyme 1, Nadp(+)-Dependent, Cytosolic* | -1.27 | -1.23 |
| *Pdha1* | *Pyruvate Dehydrogenase (Lipoamide) Alpha 1* | -1.23 | -0.44 |
| *Sdhc* | *Succinate Dehydrogenase Complex, Subunit C, Integral Membrane Protein, 15kda* | -1.21 | -0.44 |
| *Cpt2* | *Carnitine Palmitoyltransferase Ii* | -1.19 | -0.34 |
| *Pts* | *6-Pyruvoyltetrahydropterin Synthase* | -1.15 | -0.39 |
| *Gcdh* | *Glutaryl-Coenzyme A Dehydrogenase* | -1.08 | -0.56 |
| *Acat2* | *Acetyl-Coenzyme A Acetyltransferase 2 (Acetoacetyl Coenzyme A Thiolase)* | -1.05 | -0.71 |
| *Pdhb* | *Pyruvate Dehydrogenase (Lipoamide) Beta* | -1.02 | -0.59 |
| *Idh3b* | *Isocitrate Dehydrogenase 3 (Nad+) Beta* | -1.01 | -0.39 |
| *Cidea* | *Cell Death-Inducing Dffa-Like Effector A* | -0.96 | -0.50 |
| *Gpd1* | *Glycerol-3-Phosphate Dehydrogenase 1 (Soluble)* | -0.89 | -0.34 |
| *D2hgdh* | *D-2-Hydroxyglutarate Dehydrogenase* | -0.83 | -0.30 |
| *Mdh1* | *Malate Dehydrogenase 1, Nad (Soluble)* | -0.69 | -0.33 |
| *Sdhd* | *Succinate Dehydrogenase Complex, Subunit D, Integral Membrane Protein* | -0.58 | -0.57 |
| **t) Adipogenesis** | | | |
| **Gene symbol** | **Gene name** | **Rank metric score** | |
|  |  | **AT^A^** | **AO^B^** |
| *Retn* | *Resistin* | -2.82 | -1.44 |
| *Gpam* | *Glycerol-3-Phosphate Acyltransferase, Mitochondrial* | -2.53 | -0.40 |
| *Vegfb* | *Vascular Endothelial Growth Factor B* | -2.46 | -0.34 |
| *Orm1* | *Orosomucoid 1* | -2.19 | -0.84 |
| *Sult1a1* | *Sulfotransferase Family, Cytosolic, 1a, Phenol-Preferring, Member 1* | -2.17 | -0.41 |
| *Ddt* | *D-Dopachrome Tautomerase* | -2.13 | -0.45 |
| *Fah* | *Fumarylacetoacetate Hydrolase (Fumarylacetoacetase)* | -2.10 | -0.70 |
| *Mccc1* | *Methylcrotonoyl-Coenzyme A Carboxylase 1 (Alpha)* | -2.08 | -0.46 |
| *Slc1a5* | *Solute Carrier Family 1 (Neutral Amino Acid Transporter), Member 5* | -2.08 | -0.59 |
| *Bckdha* | *Branched Chain Keto Acid Dehydrogenase E1, Alpha Polypeptide* | -2.06 | -0.33 |
| *Gpd2* | *Glycerol-3-Phosphate Dehydrogenase 2 (Mitochondrial)* | -1.99 | -0.49 |
| *Dgat1* | *Diacylglycerol O-Acyltransferase Homolog 1 (Mouse)* | -1.97 | -0.74 |
| *Dbt* | *Dihydrolipoamide Branched Chain Transacylase E2* | -1.87 | -0.27 |
| *Slc25a1* | *Solute Carrier Family 25 (Mitochondrial Carrier; Citrate Transporter), Member 1* | -1.87 | -0.86 |
| *Uck1* | *Uridine-Cytidine Kinase 1* | -1.87 | -0.29 |
| *Ptger3* | *Prostaglandin E Receptor 3 (Subtype Ep3)* | -1.84 | -0.67 |
| *Adipoq* | *Adiponectin, C1q And Collagen Domain Containing* | -1.83 | -1.12 |
| *Ephx2* | *Epoxide Hydrolase 2, Cytoplasmic* | -1.82 | -0.58 |
| *Slc5a6* | *Solute Carrier Family 5 (Sodium-Dependent Vitamin Transporter), Member 6* | -1.82 | -0.73 |
| *Acly* | *Atp Citrate Lyase* | -1.81 | -1.21 |
| *Pfkfb3* | *6-Phosphofructo-2-Kinase/Fructose-2,6-Biphosphatase 3* | -1.78 | -0.76 |
| *Gbe1* | *Glucan (1,4-Alpha-), Branching Enzyme 1* | -1.76 | -0.85 |
| *Cat* | *Catalase* | -1.71 | -0.54 |
| *Cox8a* | *Cytochrome C Oxidase Subunit 8a (Ubiquitous)* | -1.71 | -0.37 |
| *Elovl6* | *Elovl Family Member 6, Elongation Of Long Chain Fatty Acids (Fen1/Elo2, Sur4/Elo3-Like, Yeast)* | -1.69 | -1.27 |
| *Adipor2* | *Adiponectin Receptor 2* | -1.60 | -0.66 |
| *Phyh* | *Phytanoyl-Coa 2-Hydroxylase* | -1.50 | -0.72 |
| *Agpat6* | *1-Acylglycerol-3-Phosphate O-Acyltransferase 6 (Lysophosphatidic Acid Acyltransferase, Zeta)* | -1.44 | -0.48 |
| *Crat* | *Carnitine Acetyltransferase* | -1.40 | -0.38 |
| *Pparg* | *Peroxisome Proliferative Activated Receptor, Gamma* | -1.40 | -0.26 |
| *Aco2* | *Aconitase 2, Mitochondrial* | -1.39 | -0.38 |
| *Slc27a1* | *Solute Carrier Family 27 (Fatty Acid Transporter), Member 1* | -1.37 | -1.23 |
| *Lama4* | *Laminin, Alpha 4* | -1.31 | -0.28 |
| *Ak2* | *Adenylate Kinase 2* | -1.29 | -0.27 |
| *Me1* | *Malic Enzyme 1, Nadp(+)-Dependent, Cytosolic* | -1.27 | -1.23 |
| *Sorbs1* | *Sorbin And Sh3 Domain Containing 1* | -1.25 | -0.57 |
| *Tst* | *Thiosulfate Sulfurtransferase (Rhodanese)* | -1.24 | -0.33 |
| *Gpx4* | *Glutathione Peroxidase 4 (Phospholipid Hydroperoxidase)* | -1.23 | -0.24 |
| *Sdhc* | *Succinate Dehydrogenase Complex, Subunit C, Integral Membrane Protein, 15kda* | -1.21 | -0.44 |
| *Cs* | *Citrate Synthase* | -1.20 | -0.37 |
| *Cpt2* | *Carnitine Palmitoyltransferase Ii* | -1.19 | -0.34 |
| *Cmbl* | *Carboxymethylenebutenolidase Homolog (Pseudomonas)* | -1.17 | -0.37 |
| *Tob1* | *Transducer Of Erbb2, 1* | -1.14 | -0.43 |
| *Idh3g* | *Isocitrate Dehydrogenase 3 (Nad+) Gamma* | -1.11 | -0.23 |
| *Cox6a1* | *Cytochrome C Oxidase Subunit Via Polypeptide 1* | -1.06 | -0.34 |
| *Gphn* | *Gephyrin* | -1.01 | -0.26 |
| *Cidea* | *Cell Death-Inducing Dffa-Like Effector A* | -0.96 | -0.50 |
| *Ywhag* | *Tyrosine 3-Monooxygenase/Tryptophan 5-Monooxygenase Activation Protein, Gamma Polypeptide* | -0.96 | -0.42 |
| *Gpx3* | *Glutathione Peroxidase 3 (Plasma)* | -0.92 | -0.48 |
| *Mrap* | *Melanocortin 2 Receptor Accessory Protein* | -0.92 | -0.80 |
| *Itga7* | *Integrin, Alpha 7* | -0.91 | -0.48 |
| *Scp2* | *Sterol Carrier Protein 2* | -0.90 | -0.76 |
| *Grpel1* | *Grpe-Like 1, Mitochondrial (E. Coli)* | -0.88 | -0.37 |
| *Sspn* | *Sarcospan (Kras Oncogene-Associated Gene)* | -0.87 | -0.28 |
| *Taldo1* | *Transaldolase 1* | -0.78 | -0.32 |
| *Cox7b* | *Cytochrome C Oxidase Subunit Viib* | -0.71 | -0.23 |
| *Agpat3* | *1-Acylglycerol-3-Phosphate O-Acyltransferase 3* | -0.70 | -0.27 |
| *Pex14* | *Peroxisomal Biogenesis Factor 14* | -0.69 | -0.22 |
| *Nkiras1* | *Nfkb Inhibitor Interacting Ras-Like 1* | -0.64 | -0.25 |
| *Ndufa5* | *Nadh Dehydrogenase (Ubiquinone) 1 Alpha Subcomplex, 5, 13kda* | -0.63 | -0.27 |
| *Prdx3* | *Peroxiredoxin 3* | -0.63 | -0.27 |
| *Samm50* | *Sorting And Assembly Machinery Component 50 Homolog (S. Cerevisiae)* | -0.61 | -0.38 |

^A^ Obese gonadal white adipose tissue; ^B^ Atherosclerotic aortae; dysregulated genes are sorted by descending rank metric score in AT.
